# A Color Image Encryption Scheme Using an Enhanced One-Dimensional Chaotic Map and Adaptive DNA Encoding

**DOI:** 10.3390/e28091015

**Published:** 2026-09-11

**Authors:** Jie Jiang, Liyuan Jiao, Yanchun Liang, Adriano Tavares, Lidong Wang

**Affiliations:** 1 School of Big Data, Zhuhai College of Science and Technology, Zhuhai 519041, China; jiangjchaos@163.com (J.J.); jiaoly1212@163.com (L.J.); 2 Department of Industrial Electronics, University of Minho, 4800-058 Guimarães, Portugal; atavares@dei.uminho.pt; 3 Faculty of Data Science, City University of Macau, Room S501, Stanley Ho Building, Avenida Padre Tomas Pereira, Taipa, Macau 410114, China; 4 School of Computer Science, Zhuhai College of Science and Technology, Zhuhai 519041, China; ycliang@jlu.edu.cn

**Keywords:** chaos theory, color image encryption, sine-tent-logistic-exponential map, adaptive position-dependent DNA encoding, authenticated encryption

## Abstract

Secure transmission and storage of color images remain challenging tasks due to strong inter-pixel correlations and high data volume. This work proposes a one-dimensional sine-tent-logistic-exponential map (STLEM) equipped with numerical boundary correction rules to mitigate finite-precision numerical degradation so as to enhance the unpredictability of chaos-driven cryptosystems. We benchmark STLEM against classic logistic, tent, and sine maps via Lyapunov exponents, autocorrelation, approximate entropy, permutation entropy, Lempel-Ziv complexity, and Kolmogorov–Sinai entropy. Bifurcation diagrams, the 0–1 test, and NIST statistical tests are further adopted to characterize its chaotic dynamics and randomness. Comparative results verify that STLEM achieves improved dynamical complexity and randomness performance. Built upon the proposed STLEM, this paper constructs a color-image encryption scheme that employs a 256-bit master key and two groups of chaotic parameters to produce key-related chaotic sequences. The cryptosystem integrates dynamic edge expansion, chaotic permutation, position-dependent adaptive DNA encoding, DNA-domain chained diffusion, and two successive row-column permutation phases. HMAC-SHA-256 is utilized to generate plaintext-aware initial conditions and perform ciphertext authentication prior to decryption. Experimental validations demonstrate complete plaintext recovery under valid secret inputs, while authentication rejects invalid keys and tampered ciphertexts. Ciphered images exhibit high information entropy, negligible adjacent-pixel correlations, and satisfactory number of pixel change rate (NPCR) and unified average changing intensity (UACI) metrics. Benefiting from a sufficiently large key space and O(MNlog(MN)) computational complexity, the proposed scheme is resilient against brute-force attacks and well suited for secure color-image communication scenarios, rather than acting as a general-purpose replacement for standard block ciphers.

## 1. Introduction

The digital revolution has greatly promoted the development of multimedia technology. Beyond the traditional function of information presentation, images now carry core data for military communication, medical diagnosis and public security fields. Once satellite images containing troop deployment information are intercepted during transmission, or patients’ MRI data is tampered with before being sent to clinicians, severe risks will be triggered. Therefore, guaranteeing the security of image transmission has become increasingly important.

Conventional image encryption techniques generally have unsatisfactory security performance. To address this issue, researchers in information security have introduced diverse technologies to enhance the security of image encryption. In 1998, J. Fridrich pioneered the application of chaotic systems to image encryption [1].

On this basis, image encryption has developed rapidly, and numerous novel algorithms have been proposed, including schemes based on deoxyribonucleic acid (DNA) encoding [2], wavelet transform [3] and genetic recombination [4]. Most of these methods combine chaotic systems with different encryption frameworks and carry out targeted optimizations. At present, designing high-performance image encryption algorithms by integrating chaotic systems with other technologies has become a popular research trend. As practical requirements become more stringent, traditional one-dimensional chaotic maps fail to satisfy the security demands of modern image encryption. For this reason, scholars have focused on modifying existing chaotic maps and enhancing their chaotic dynamics for encryption applications [5,6,7,8,9,10,11,12,13]. Niu et al. [14] designed an improved two-dimensional Hénon map and a modified one-dimensional logistic map. By optimizing the classic models, the original Hénon map was transformed into an effective encryption primitive. Ding et al. [15] proposed a reversible image encryption algorithm based on a new three-dimensional logistic map (3D-LM), which achieves efficient data scrambling. Sun et al. [16] presented a multi-layer encryption scheme that combines the logistic-sine chaotic map (LSCM) with cellular automata to improve overall security. Wang et al. [17] established a two-dimensional logarithmic-logarithmic sine chaotic map (2D-LLSCM) based on nonlinear logarithmic functions, aiming to improve the dynamic complexity of chaotic sequences. The adopted nonlinear function effectively increases the nonlinear characteristics of the chaotic system. Gao et al. [18] proposed a parallel color image encryption algorithm using the 2D Logistic-Rulkov neuron map (2D-LRNM), which organically integrates neural networks and chaotic systems. Xu et al. [19] constructed a quantum logistic-tent map and extended it to a fractional-order version. They investigated the nonlinear behaviors of the map using bifurcation diagrams, Lyapunov exponent spectra and spectral entropy. The experimental results indicate that the fractional-order map has stronger nonlinearity and better applicability to image encryption.

Traditional image encryption algorithms usually offer only a layer of security. As the underlying computational process becomes public, the confidentiality of the data may be compromised. DNA cryptography utilizes biological and computational properties to provide greater confidentiality than standard cryptographic algorithms when encrypting data [20]. DNA cryptography takes advantage of the self-assembly properties of DNA bases and combines them with cryptographic techniques to offer multiple security measures, thereby enhancing the degree of realization of data confidentiality [21]. For example, the authors in [22] utilized the amino acid table to convert the ciphertext to genomic form. The protein sequences in these tables increased the uncertainty level of the ciphertext. In recent years, due to the numerous excellent characteristics of DNA computing, such as its great parallelism, massive storage capacity, and low power consumption, it has been applied to image chaotic cipher systems [23,24,25,26,27,28,29,30].

In 2016, Kulsoom et al. [31] extracted the most significant and least significant bits of each pixel and carried out DNA operations. The proposed method exhibited poor anti-noise performance as the majority of pixel values remained unaltered. In 2018, Wei Ran et al. further developed an image encryption system that integrates DNA encoding with multiple chaotic maps [32]. Experimental results verified that the scheme effectively boosted plaintext and key sensitivity. Nevertheless, it suffers from high computational complexity and strict hardware requirements. Gao et al. [33] put forward a flexible multi-image encryption scheme based on a new n-dimensional chaotic model and octal DNA operations. The scheme supports the encryption of images with arbitrary categories, numbers and resolutions. With the help of customized key generation and SCAN-based scrambling, the overall security and anti-attack ability are significantly enhanced. Qiu et al. [25] devised an advanced image encryption algorithm combining DNA S_boxes and hyperchaotic systems. It dynamically constructs DNA S_boxes via 2-bit DNA encoding and 4-bit quasi-DNA encoding, and enables seamless transformation between the two encoding strategies. As revealed by experimental results, the key space reaches around $10^{145}$, the resistance to differential attacks is over 99.5%, and the information entropy of the ciphertext exceeds 7.997.

The core drawback of traditional one-dimensional chaotic maps for image encryption stems from three critical limitations. First, they possess narrow, discontinuous chaotic ranges. The classic logistic map, for instance, only exhibits chaotic dynamics when μ∈[3.57,4], leaving an extremely limited parameter space. Second, their output sequences follow severely non-uniform distributions, with most values clustered at the interval boundaries. Such biased statistical properties depart from ideal randomness and render the cryptosystem susceptible to statistical analysis attacks. Third, single maps feature overly simplistic structures; the generated pseudorandom sequences lack sufficient nonlinear complexity, which yields a tiny key space and unsatisfactory security performance, thus failing to withstand brute-force and known-plaintext attacks. Motivated by the above limitations, this paper proposes further improvements detailed in the subsequent two sections.

Firstly, a new one-dimensional chaotic system is constructed by integrating the features of the sine map, tent map and logistic map. Compared with conventional one-dimensional chaotic maps, this system has a wider key space and better statistical characteristics. Secondly, we combine the improved chaotic sequence with dynamic DNA encoding to establish the proposed “living encryption” framework.

In summary, the present study delivers three key research contributions. First, we develop an enhanced one-dimensional STLEM chaotic map that merges nonlinear terms from sine, tent, logistic and exponential functions. When benchmarked against classic one-dimensional chaotic counterparts, STLEM yields a broader stable chaotic-parameter interval, higher Lyapunov exponent values, and improved randomness behaviors. We further carry out systematic analysis to examine side effects brought by modulo arithmetic, boundary clipping, and numerical-reset operations, particularly for real-world finite-precision computation scenarios. Second, we realize a complete authenticated color-image encryption architecture on top of STLEM. This framework integrates dynamic edge-redundancy processing, position-dependent adaptive DNA encoding, DNA-domain chained CBC-like diffusion, alongside two-stage row-column permutation. Unlike many reported DNA-chaos cryptosystems that stick to static global DNA coding rules, our method produces spatially varying DNA rules from chaotic outputs without bringing extra metadata overhead. Third, HMAC-SHA-256 is deeply incorporated within chaotic-seed derivation workflows. This enables plaintext-aware seed computation and built-in ciphertext authentication. The authentication function filters out tampered ciphertext and invalid secret inputs before any decryption step, a capability rarely realized within most published chaos-DNA image encryption works.

## 2. The Proposed Improved Hybrid Chaotic Map

### 2.1. Sine-Tent-Logistic-Exponential Map (STLEM)

First, we return to the classic sine map, tent map and logistic map. Then, based on these three maps, we make improvements and obtain a new chaotic map: sine-tent-logistic-exponential map (STLEM). The mathematical formula of the one-dimensional logistic map is given in Equation (1):

(1) $$x_{n+1}=rx_{n}\left(1-x_{n}\right)$$

where *r* represents the control parameter. This map behaves chaotically when r∈(3.57,4]. The mathematical expression of the sine chaotic map is as shown in Equation (2):

(2) $$x_{n+1}=\frac{a}{4}sin\left(\pi x_{n}\right)$$

where *a* is the control parameter, and the chaotic interval is (1, 2]. The mathematical formula of the tent map is presented in Equation (3):

(3) $$x_{n+1}=\left\{\begin{array}{cc} rx_{n}, & \mathrm{if}\;x_{n}\leq 0.5,\\ r(1-x_{n}), & \mathrm{if}\;x_{n}>0.5 \end{array}\right.$$

where *r* denotes the control parameter, and the chaotic interval is (1,2].

Considering the complexity requirements of image encryption, this paper fuses the above three classical chaotic maps and proposes an improved one-dimensional chaotic map, named the sine-tent-logistic-exponential map (STLEM). The mathematical expression of the STLEM is defined in Equations (4) and (5):

(4) $$x_{n+1}=\left\{\begin{array}{cc} F_{1}\left(x_{n}\right), & \mathrm{if}\;x_{n}\leq 0.5\\ F_{2}\left(x_{n}\right), & \mathrm{if}\;x_{n}>0.5 \end{array}\right.$$

where

(5) $$\left\{\begin{array}{c} \begin{array}{c} F_{1}\left(x_{n}\right)=\mathrm{mod}(ax_{n}+\left(8-a\right)\sin\left(\pi x_{n}\right)+bx_{n}^{3}+ce^{dx_{n}},\;1), \end{array}\\ \begin{array}{cc} F_{2}\left(x_{n}\right)=\mathrm{mod}(a\left(1-x_{n}\right)+\left(8-a\right)\sin\left(\pi \left(1-x_{n}\right)\right)\; & +b{\left(1-2x_{n}\right)}^{3}+ce^{d(1-x_{n})},\;1), \end{array}\\ x_{n}=0.5,\;\mathrm{if}\;x_{n}\;\mathrm{is}\;\mathrm{NaN}\;\mathrm{or}\;\infty ,\\ x_{n+1}=0.48,\;\mathrm{if}\;x_{n+1}<10^{-12},\\ x_{n+1}=0.52,\;\mathrm{if}\;x_{n+1}>1-10^{-12}. \end{array}\right.$$

where *a*, *b*, *c* and *d* denote control parameters. To guarantee the numerical stability of chaotic iteration, several auxiliary operations are adopted in this work. First, the input state is normalized by a modulo 1 operation to confine the value within the interval [0,1). If the iteration state becomes infinite or non-numeric (NaN), it will be reset to 0.5 to maintain the continuity of iteration. Furthermore, to avoid the system falling into fixed points near the boundaries, the output value of the chaotic map calculated by Equation (4) is further constrained: it is adjusted to 0.48 when the value is less than $10^{-12}$, and set to 0.52 when the value exceeds $1-10^{-12}$. These strategies effectively prevent iteration degradation and ensure the ergodicity and randomness of the generated chaotic sequences. By integrating the logistic map, sine map and tent map and introducing exponential nonlinearity, the proposed system achieves higher complexity, a wider parameter range and better randomness.

### 2.2. Lyapunov Exponent Analysis

The Lyapunov exponent (LE) is widely adopted to evaluate the sensitivity of dynamical systems to initial values. It characterizes the average divergence speed of nearby trajectories within the phase space. A positive LE demonstrates that the system is chaotic, while a negative or zero LE indicates stability or periodicity. The calculation formula for the Lyapunov exponent is given in Equation (6):

(6) $$\lambda =\lim_{N\to \infty }\frac{1}{N}\sum_{n=0}^{N-1}\ln\left|\frac{df(x_{n})}{dx}\right|$$

where *n* denotes the index of the data points in the generated time series $f(x_{n})$.

**Figure 1:**
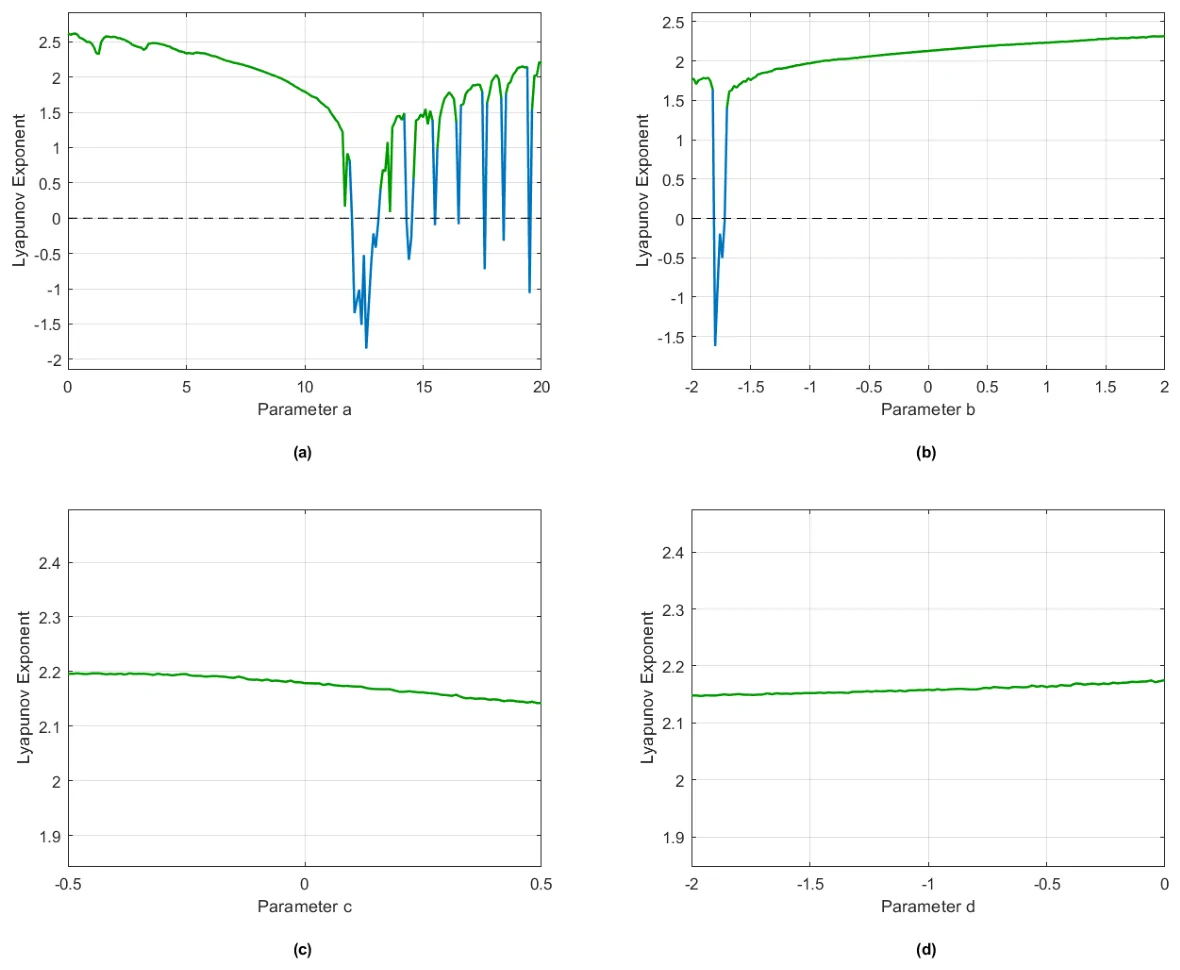
(**a**–**d**) show the Lyapunov exponent diagrams corresponding to parameters *a*, *b*, *c* and *d*, respectively. The blue curves represent the full largest Lyapunov exponent across all sampled parameters. Green curves overlay only points with positive values to denote chaos; in oscillatory regions, positive peaks are green, and negative troughs remain blue.

For reproducibility, the reference configuration for all non-swept parameters is $\left[a,b,c,d\right]=\left[7.5,0.3,0.2,-0.5\right]$ with initial condition $x_{0}=0.3$. The one-parameter sweeps adopt 201, 201, 101, and 101 uniformly-spaced sampling points for a∈[0,20], b∈[−2,2], c∈[−0.5,0.5], and d∈[−2,0], respectively. All iterates are computed under IEEE-754 [34] double-precision floating-point arithmetic. For each parameter sample, the first 2000 iterations are discarded as transient evolution, and the following 10,000 iterations are utilized to calculate the Lyapunov exponent according to Equation (6). The system state is taken modulo one following the definition of the map. An optional near-boundary correction rule is implemented and logged during computation; this correction is never triggered across all points of the Lyapunov-exponent sweeps, and therefore no boundary reset affects the numerical results shown in Figure 1.

The Lyapunov exponent (LE) curves of the proposed map are illustrated in Figure 1. Most parameter regions yield predominantly positive LE values, demonstrating that the system can sustain chaotic dynamics over wide parameter spaces. For parameter *a* spanning from 0 to 20, the LE starts at a large positive value and gradually decreases with increasing *a*. Positive LE values corresponding to chaotic behavior are observed for a∈[0,12.0]. Narrow periodic windows with negative LE emerge around a∈[12.0,13.0]. Within a∈[13.0,20.0], the LE fluctuates between positive and negative values, indicating alternating chaotic and periodic dynamical states. For parameter *b*, negative LE only occurs within an extremely narrow interval near b=−2, while the LE remains stably positive in the majority of the investigated range b∈(−1.8,2.0], varying between 2.0 and 2.3. For parameters *c* and *d*, the LE curves stay consistently positive across the entire scanned parameter domains without observable periodic windows. The widespread positive LE characteristics confirm that the proposed map can generate highly unpredictable chaotic sequences, which is essential for constructing secure image-encryption schemes.

### 2.3. Analysis of Bifurcation Diagrams

To comprehensively investigate the parameter-dependent dynamical evolution of the newly constructed chaotic map, we first adopt bifurcation diagrams to visualize the iterative state variation trajectories as single control parameters change, and then we further utilize Lyapunov exponent (LE) heat maps to quantify the chaotic intensity under two-parameter coupling scenarios.

For each one-dimensional bifurcation diagram, uniformly-spaced parameter samples are adopted over the same intervals as in Section 2.2, while the remaining parameters are kept fixed at $\left[a,b,c,d\right]=\left[7.5,0.3,0.2,-0.5\right]$ with initial condition $x_{0}=0.3$. For each parameter value, the first 2000 iterations are discarded as transient, and the subsequent 500 state points are rendered for visualization. The logged near-boundary correction counter remains zero for all sampled parameters. Consequently, the presented orbits are generated purely by the modulo-defined map, and no near-boundary reset is activated under this numerical protocol.

**Figure 2:**
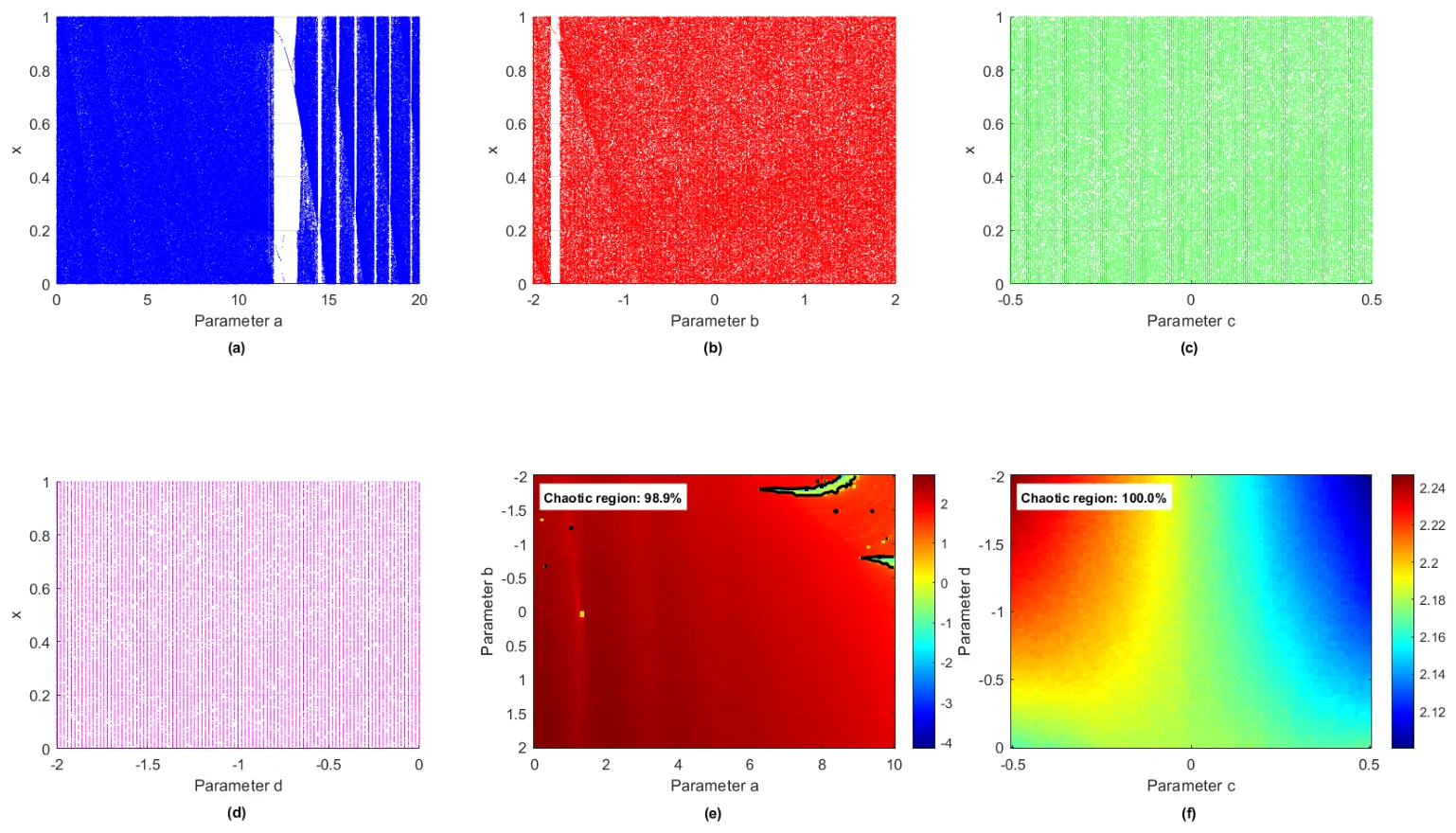
(**a**–**d**) display the bifurcation diagrams of parameters *a*, *b*, *c* and *d*, while (**e**,**f**) illustrate the Lyapunov exponent heat maps for parameter pairs *a*–*b* and *c*–*d*, respectively.

The visualized results are presented in Figure 2, where subplots (a)–(d) depict the single-parameter bifurcation behaviors for parameters *a*, *b*, *c*, and *d* respectively, and subplots (e)–(f) render the LE heat maps for the parameter pairs *a*–*b* and *c*–*d* to characterize multi-parameter coupling dynamics. Detailed observations and interpretations of these graphical results are elaborated as follows.

As illustrated in Figure 2, the bifurcation diagrams clearly reveal the dynamical behavior of the proposed chaotic map. For a∈[0,12.0], iterative state values spread densely and uniformly over the full interval x∈[0,1], exhibiting favorable ergodic properties. Consistent with the LE results, obvious alternating chaotic and periodic behavior occurs for a>12.0. For parameter *b*, a tiny periodic gap emerges only in the immediate vicinity of b=−2, whereas the bifurcation diagram is filled with dense scattered points across the remaining parameter range. In contrast, the bifurcation diagrams of parameters *c* and *d* are fully covered by uniform state points throughout the entire parameter range, with no visible periodic or blank regions.

These phenomena demonstrate that the proposed chaotic system possesses a wide and robust chaotic parameter range. The vast majority of parameter combinations can maintain stable chaotic states, effectively avoiding predictable periodic or quasi-periodic behaviors that may cause security vulnerabilities in encryption applications. Furthermore, the chaotic orbits are evenly distributed within [0,1] without obvious aggregation or voids, indicating that the generated pseudo-random sequences have outstanding uniform distribution characteristics.

The Lyapunov exponent heat maps intuitively and quantitatively reveal the chaotic performance of the proposed map under multi-parameter coupling conditions, and we separately analyze the two groups of heat maps, i.e., the *a*–*b* coupling heat map and the *c*–*d* coupling heat map. For the *a*–*b* Lyapunov exponent heat map, the color bar shows that the Lyapunov exponent values of valid chaotic regions range from 1.6 to 2.3. Massive deep-red areas correspond to LE values higher than 2.0, which demonstrates strong chaotic intensity of the coupled *a* and *b* parameters. Consistent with the single-parameter LE curves and bifurcation diagrams, sparse local blue low-value non-chaotic regions are observed over the heat map, corresponding to periodic windows at large *a* values and the narrow non-chaotic neighborhood near b=−2. As marked in Figure 2, the chaotic region accounts for 98.9% of the entire *a*–*b* parameter plane, which proves that the two control parameters possess flexible, robust chaotic coupling characteristics and a huge available key space for encryption. In contrast, the *c*–*d* heat map exhibits smooth, uniform color distribution without any dark blue low-value or blank non-chaotic areas. All combinations of *c* and *d* produce steady positive Lyapunov exponents between 2.15 and 2.22. This result fully verifies that *c* and *d* can maintain persistent, stable and strong chaotic dynamics across the whole tested range, free of periodic interference. Overall, the majority of parameter combinations yield high positive LE values, which confirms the system’s extreme sensitivity to initial values and tiny parameter perturbations. The gradual color gradient in both heat maps visually reflects how chaotic intensity evolves with coupled parameter variations, which provides a reliable experimental basis for selecting high-security parameter groups and constructing high-quality pseudo-random key streams for image encryption.

To further validate the intrinsic dynamical characteristics and numerical robustness of the proposed STLEM map, the influences of internal modulo operation, exponential nonlinear terms, boundary constraints, and numerical reset strategies on system behavior are systematically discussed. Firstly, the modulo-one operation strictly restricts all iterative states within [0,1], producing bounded orbits and eliminating divergent iterations. This canonical operation complies with the general design principle of discrete chaotic maps and introduces no artificial distortion to the inherent dynamics. Secondly, the hybrid exponential and polynomial nonlinearities significantly enrich the system’s dynamical complexity, contributing to high Lyapunov exponents and uniform orbital distribution, which effectively improves the randomness and ergodicity compared with conventional low-dimensional chaotic systems.

Thirdly, the embedded near-boundary correction and numerical reset rules are specially designed to eliminate computational singularities, including NaN and infinite values, during IEEE-754 double-precision numerical iterations. Notably, the correction mechanism is never activated throughout all Lyapunov exponent calculations and bifurcation simulations in this work, with a zero activation count for all sampling points. This indicates that all observed bifurcation structures, periodic windows, and Lyapunov exponent characteristics are completely generated by the inherent iterative formula of the STLEM map rather than through external artificial intervention.

For secure key generation in image encryption applications, we derive conservative chaotic parameter intervals that exclude large contiguous periodic patches and pre-periodic transition zones. Within these intervals, the maximum Lyapunov exponent stays persistently positive without obvious periodic windows. Based on the bifurcation diagrams, one-dimensional Lyapunov-exponent curves, and two-dimensional LE heat-maps, the conservative chaotic ranges are given as a∈(0,9.0], b∈(−1.6,2.0], c∈[−0.5,0.5], and d∈[−2.0,0]. It should be emphasized that the range 9.0<a≤12.0 corresponds to a pre-periodic transition zone containing intermittent narrow periodic windows. Although chaotic behavior can still be observed for some sampling points within this zone, coexisting periodic components introduce potential security threats; therefore, this zone is excluded from the conservative parameter set. Inside the proposed conservative intervals, the system yields high positive Lyapunov exponents and strong chaotic dynamics, and large-scale periodic regions are fully avoided. The valid key space for encryption is constructed mainly within these conservative intervals, which provides solid dynamical guarantees for the subsequent cryptographic experiments.

### 2.4. Dynamical Analysis via the 0–1 Test

The 0–1 test for chaos was employed to independently assess whether the proposed STLEM exhibits chaotic rather than regular periodic or quasi-periodic dynamics. In contrast to the maximal Lyapunov exponent, which quantifies the long-term average separation rate of nearby trajectories, the 0–1 test operates directly on a scalar time series and characterizes the growth of auxiliary translation variables.

Let ${\left\{x_{n}\right\}}_{n=1}^{N_{s}}$ denote the STLEM orbit after discarding transient iterations. For each frequency parameter θ∈Θ⊂(0,π), the translation variables are defined by

(7) $$\left\{\begin{array}{c} p_{\theta }\left(n\right)=\sum_{j=1}^{n}x_{j}\cos\left(j\theta \right),\\ q_{\theta }\left(n\right)=\sum_{j=1}^{n}x_{j}\sin\left(j\theta \right), \end{array}\right.$$

where Θ denotes a set of sampled frequencies chosen away from 0 and π to mitigate numerical resonance effects.

For a finite sequence, the mean squared displacement is calculated as

(8) $$M_{\theta }\left(n\right)=\frac{1}{N_{s}-n}\sum_{j=1}^{N_{s}-n}\left\{{\left[p_{\theta }\left(j+n\right)-p_{\theta }\left(j\right)\right]}^{2}+{\left[q_{\theta }\left(j+n\right)-q_{\theta }\left(j\right)\right]}^{2}\right\}.$$

Because the generated orbit generally has a nonzero mean, the modified mean squared displacement is expressed as

(9) $$D_{\theta }\left(n\right)=M_{\theta }\left(n\right)-V_{\mathrm{osc}}\left(\theta ,n\right),$$

where

(10) $$V_{\mathrm{osc}}\left(\theta ,n\right)={\bar{x}}^{\;2}\frac{1-\cos(n\theta )}{1-\cos(\theta )},\;\bar{x}=\frac{1}{N_{s}}\sum_{j=1}^{N_{s}}x_{j}.$$

The correlation-coefficient version of the 0–1 test was adopted. Specifically, for $n=1,\ldots ,n_{\mathrm{cut}}$, with $n_{\mathrm{cut}}\ll N_{s}$, the statistic for each sampled frequency is

(11) $$K\left(\theta \right)=\mathrm{corr}\left(\left[1,2,\ldots ,n_{\mathrm{cut}}\right],\left[D_{\theta }\left(1\right),D_{\theta }\left(2\right),\ldots ,D_{\theta }\left(n_{\mathrm{cut}}\right)\right]\right).$$

The robust test statistic is then obtained as

(12) $$K_{\mathrm{med}}={\mathrm{median}}_{\theta \in \Theta }\left\{K\left(\theta \right)\right\}.$$

A value of $K_{\mathrm{med}}$ close to zero indicates regular motion, whereas a value close to unity provides evidence of chaotic dynamics. The reference line K=0.97 used in the figures is a conservative empirical acceptance criterion and is not a universal threshold of the 0–1 test.

All computations employ IEEE-754 double-precision arithmetic under MATLAB R2020a. The reference parameter vector is $\left[a,b,c,d\right]=\left[7.5,0.3,0.2,-0.5\right]$. Four distinct initial states, $x_{0}\in \left\{0.3,0.314159265,0.718281828,0.901234567\right\}$, are evaluated for every parameter vector. The full reproducible numerical protocol is summarized in Table 1. In particular, the 64 quasi-random parameter vectors are generated via a scrambled Sobol low-discrepancy sequence with rng(20260817,’twister’) fixed prior to orbit generation; no parameter configuration is manually selected post hoc to favor desirable chaotic outcomes.

A conservative reset-type boundary handling policy is activated as a fail-safe mechanism: whenever an iterate falls below $10^{-12}$ or above $1-10^{-12}$, it is reassigned to 0.48 or 0.52, respectively, and the correction event is recorded. Across all 68×4=272 trajectories, the total number of boundary correction events is **zero**; that is, no trajectory ever invokes the reset clause. This observation is significant for two reasons. First, the STLEM map remains naturally confined within [0,1] throughout the explored parameter domain, so the observed dynamics are intrinsic to the map rather than artifacts of artificial state re-injection. Second, because no reset occurs, every finite-time Lyapunov exponent is computed purely from the analytic derivative of the map along the orbit, with no numerical intervention that could inflate or distort the separation rate. The zero-correction result therefore strengthens the interpretation that positive Lyapunov exponents and K→1 are genuine properties of the STLEM dynamics.

**Figure 3:**
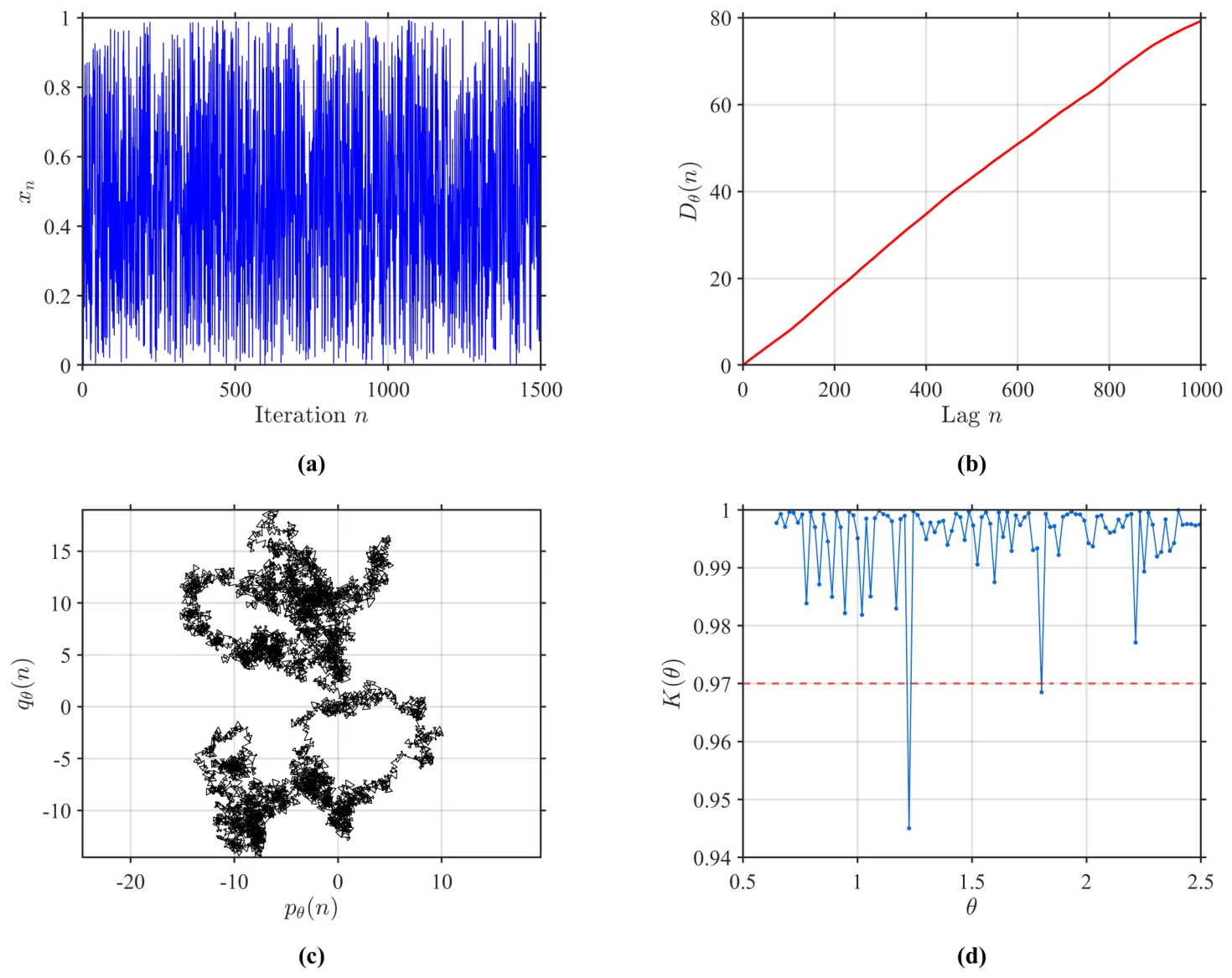
Representative 0–1 test for the fixed STLEM parameter vector. (**a**) Post-transient orbit; (**b**) modified mean-square displacement $D_{\theta }\left(n\right)$ at θ=2.17734; (**c**) translation-variable trajectory; (**d**) K(θ) over the 100 predetermined frequencies. The red dashed line at 0.97 is an empirical visual reference, not a universal decision threshold. Results are shown for initial state $x_{0}=0.3$; the robust result for this orbit is $K_{\mathrm{med}}=0.997669$.

Figure 3a shows that the post-transient orbit varies irregularly throughout the interval [0,1], without an apparent repeating pattern. For the representative frequency θ=2.17734, Figure 3b exhibits an approximately linear increase in $D_{\theta }\left(n\right)$, which is consistent with the diffusive growth expected for chaotic dynamics. The translation-variable trajectory in Figure 3c forms an irregular, non-closed two-dimensional excursion rather than a bounded periodic loop.

For this reference parameter vector, four distinct initial states are tested: $x_{0}=0.3$, 0.314159265, 0.718281828, and 0.901234567. The four orbit-wise robust $K_{\mathrm{med}}$ statistics obtained from these four initial states are 0.997669, 0.997557, 0.997958, and 0.998163. Their median across initial conditions equals 0.997814, and the corresponding mean maximal Lyapunov exponent is 2.164784. The orbit visualized in Figure 3 corresponds to the first initial condition $x_{0}=0.3$, yielding $K_{\mathrm{med}}=0.997669$. As shown in Figure 3d, most individual K(θ) values remain close to unity, although isolated frequency values may yield lower statistics. Such isolated drops are known numerical artifacts of the 0–1 test arising at certain resonant frequencies. Accordingly, we adopt the median-aggregated statistic $K_{\mathrm{med}}$ over all sampled frequencies as our robust measure, instead of interpreting individual K(θ) values.

**Figure 4:**
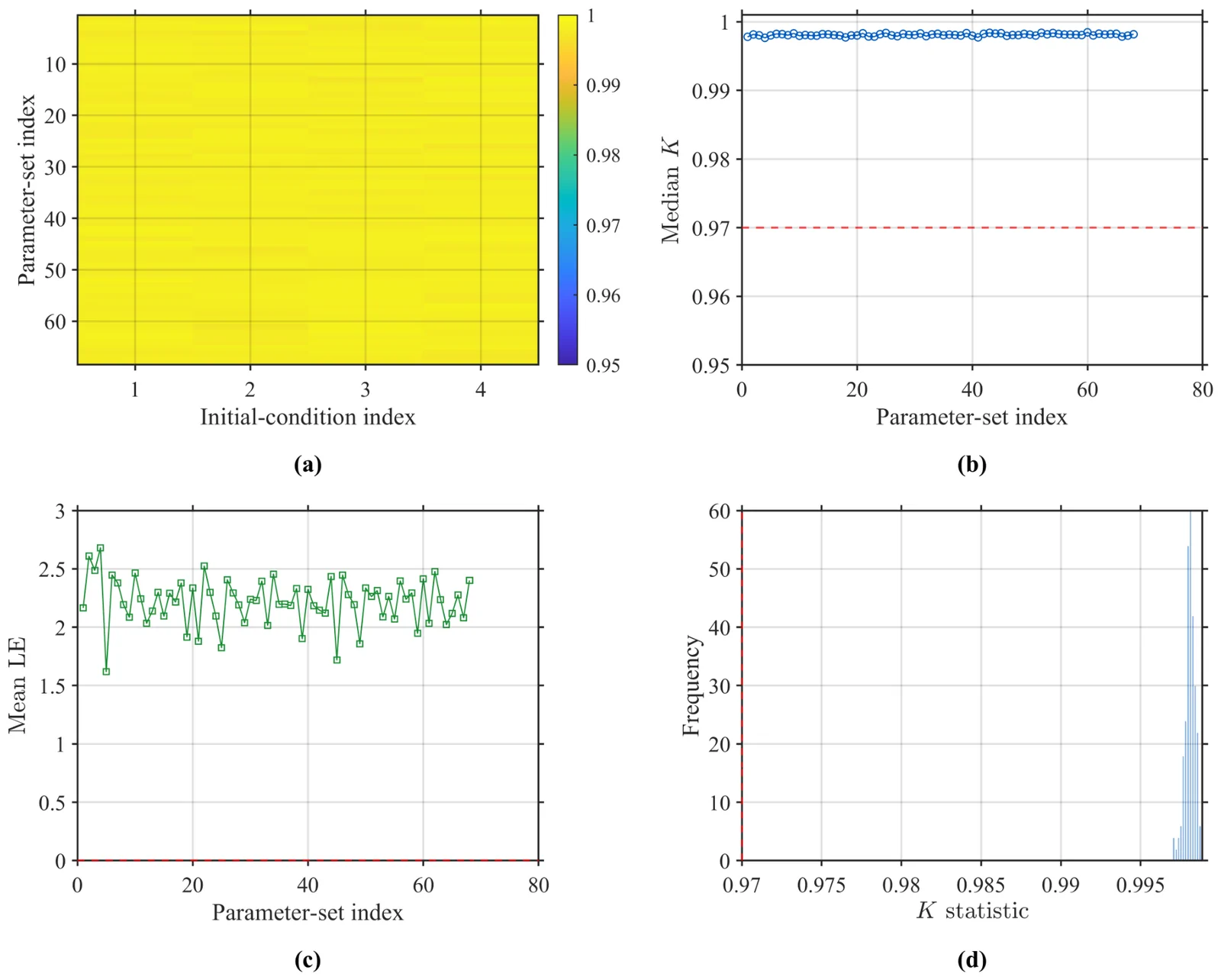
Cross-validation of the 0–1 test and maximal Lyapunov exponents across 68 STLEM parameter sets with four initial conditions. (**a**) Robust $K_{\mathrm{med}}$ values for all 272 parameter–initial-condition pairs visualized as heatmap; (**b**) median robust $K_{\mathrm{med}}$ for each parameter set; (**c**) mean maximal Lyapunov exponent for each parameter set; (**d**) histogram of all 272 robust $K_{\mathrm{med}}$ values. The red dashed line marks the empirical reference level K=0.97.

To examine robustness across the parameter space, cross-validation was conducted over 68 parameter vectors and four initial-condition realizations per parameter set, yielding 68×4=272 robust $K_{\mathrm{med}}$ statistics in total. Figure 4a shows that the robust *K* values are concentrated in a narrow high-value range for all the pairs of initial conditions tested. Consistently, Figure 4b demonstrates that the median robust statistic of every parameter set remains well above the conservative reference level K=0.97 and close to unity.

Figure 4c presents the corresponding mean maximal Lyapunov exponents. All parameter sets yield strictly positive mean values, spanning [1.619983,2.680727], providing an independent dynamical indicator consistent with the 0–1 test. The histogram in Figure 4d further confirms that the computed robust *K* values cluster close to unity rather than near the regular dynamics limit.

Across all 272 evaluated trajectories, $K_{\mathrm{med}}$ lies within [0.997035,0.998764] with an overall sample mean of 0.998096. After aggregating to the parameter-set level by taking the median across four initial-condition realizations, parameter-wise $K_{\mathrm{med}}$ ranges from 0.997669 to 0.998467. Every evaluated parameter set satisfies the descriptive criteria $K_{\mathrm{med}}\geq 0.97$ and $K_{q25}\geq 0.97$, where $K_{q25}$ denotes the lower quartile computed across the four orbit-wise robust statistics for a given parameter vector.

Overall, the 0–1 test and the positive maximal Lyapunov exponents provide mutually consistent numerical evidence that the STLEM remains chaotic within the investigated parameter and initial-condition domain. Notably, zero boundary-correction events were observed across all 272 trajectories, confirming that the reported chaotic signatures are not artifacts induced by artificial state re-injection.

### 2.5. Comparative Analysis

To quantitatively evaluate the chaotic behaviors of the proposed STLEM, four widely recognized metrics, including the Lyapunov exponent, permutation entropy (PE), approximate entropy (ApEn), and normalized Lempel–Ziv complexity, are adopted. Meanwhile, the autocorrelation function is analyzed to characterize the linear dependence of the generated time series. The conventional sine map, tent map, and logistic map are employed as benchmark models for comparison.

Numerical evaluations are conducted with initial condition $x_{0}=0.3$. A burn-in transient of 2000 iterations is discarded to eliminate transient effects, and a subsequent sequence of 20,000 points is generated for metric computation. For permutation entropy, the embedding dimension is set to m=5 and time delay τ=1. For approximate entropy, we use embedding dimension m=2 and tolerance threshold r=0.2·σ, where σ denotes the standard deviation of the time-series segment, with a sampled length of 3000. The Lempel–Ziv complexity is computed on a binary sequence obtained by median-threshold binarization, and autocorrelation coefficients are calculated up to a maximum lag of 50.

**Figure 5:**
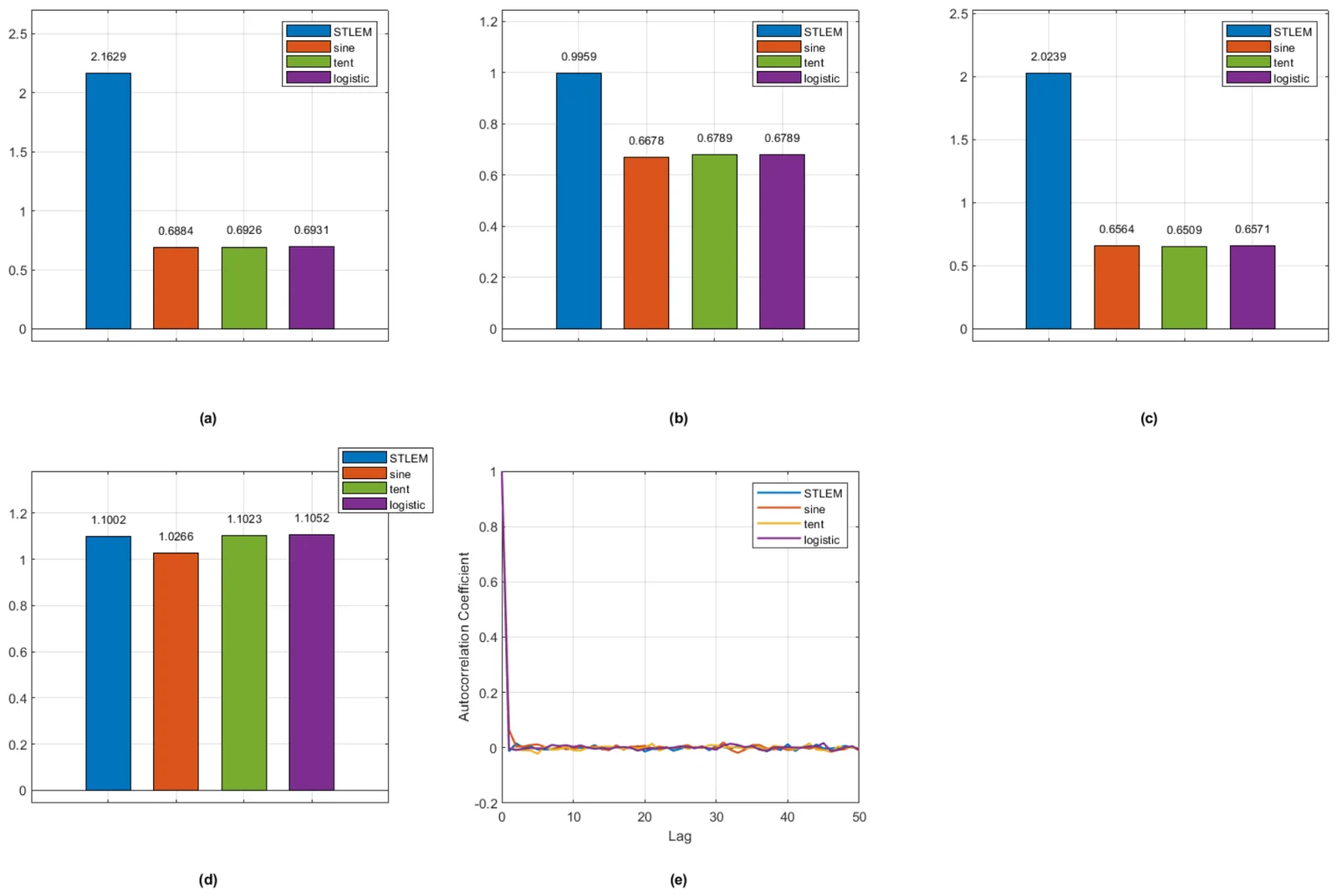
Performance comparison of STLEM, sine map, tent map and logistic map. (**a**) Lyapunov exponent. (**b**) Permutation entropy. (**c**) Approximate entropy. (**d**) Normalized Lempel–Ziv complexity. (**e**) Autocorrelation function.

The Lyapunov exponent (LE) quantitatively describes the sensitivity of a chaotic system to initial values. A positive LE value indicates chaotic behavior, and a larger LE corresponds to faster divergence of adjacent orbits and stronger unpredictability. As shown in Figure 5a, the LE value of STLEM reaches 2.1629, which is considerably higher than those of the sine map (0.6884), tent map (0.6926), and logistic map (0.6931). This result demonstrates that STLEM exhibits more remarkable sensitivity to initial conditions and superior chaotic dynamical properties.

Permutation entropy quantifies the ordinal-pattern distribution of chaotic sequences. It prevents the generated keystream from falling into periodic or quasi-periodic states, which would otherwise produce weak keys and degrade encryption security. The calculation formula of PE is defined as follows:

(13) $$H\left(m\right)=-\sum_{j=1}^{K}p_{j}\ln p_{j},\;H_{\mathrm{norm}}=\frac{H(m)}{\ln(m!)}$$

where *m* denotes the embedding dimension that determines the length of each reconstructed ordinal pattern, and τ represents the time delay between adjacent elements of the reconstructed vector. *K* is an integer satisfying K≤m!, representing the total number of distinct ordinal patterns observed in the sequence, while $p_{j}$ corresponds to the occurrence probability of the *j*-th ordinal pattern. The normalized entropy $H_{\mathrm{norm}}$ scales the PE value into the range [0,1], where 0 corresponds to a completely regular sequence and 1 indicates an ideal random sequence. Permutation entropy measures the randomness and disorder of time series; higher PE values indicate weaker sequence predictability. It can be observed from Figure 5b that the permutation entropy of STLEM is 0.9959, which significantly outperforms the sine map (0.6678), tent map (0.6789), and logistic map (0.6789).

Approximate entropy characterizes the regularity and predictability of chaotic trajectories, where a higher ApEn value indicates superior sequence irregularity and better resistance against statistical-prediction attacks. The ApEn formula is expressed as:

(14) $$\left\{\begin{array}{c} ApEn\left(m,r,N\right)=\Phi^{m}\left(r\right)-\Phi^{m+1}\left(r\right)\\ \Phi^{m}\left(r\right)=\frac{1}{N-m+1}\sum_{i=1}^{N-m+1}\ln C_{i}^{m}\left(r\right)\\ C_{i}^{m}\left(r\right)=\frac{\#\{d_{m}\left[X\left(i\right),X\left(j\right)\right]<r\}}{N-m+1}\\ d_{m}\left[X\left(i\right),X\left(j\right)\right]=\max_{k=0,\ldots ,m-1}\left|u\left(i+k\right)-u\left(j+k\right)\right| \end{array}\right.$$

where *N* is the length of the time series, *m* is the embedding dimension, and *r* is the tolerance threshold. Generally, *r* is set to 0.2 times the standard deviation of the sequence to judge the similarity between two reconstructed vectors. The Chebyshev distance $d_{m}\left[X\left(i\right),X\left(j\right)\right]$ is adopted to calculate the maximum absolute difference between corresponding elements of two *m*-dimensional vectors. $C_{i}^{m}\left(r\right)$ represents the proportion of vectors whose distance from the *i*-th template vector X(i) is smaller than the threshold *r*, and $\Phi^{m}\left(r\right)$ denotes the average logarithmic matching rate of all template vectors. A smaller ApEn value implies higher sequence regularity and lower randomness. The approximate-entropy results illustrated in Figure 5c lead to consistent conclusions. The approximate entropy of STLEM achieves 2.0239, while the values of the three classic chaotic maps are all below 0.66. The above two entropy metrics jointly verify that the time series generated by STLEM possesses greater irregularity and better randomness.

Lempel–Ziv complexity assesses the compressibility of chaotic sequences; a normalized Lempel–Ziv complexity closer to unity indicates that the keystream approximates a purely random binary sequence and can effectively resist compression-based distinguishing attacks. The mathematical formula for normalized LZ complexity is given as follows:

(15) $$\left\{\begin{array}{c} C\left(n\right)=\frac{c\left(n\right)\;\cdot \;\log_{2}n}{n},\\ C_{\mathrm{norm}}=\frac{C(n)}{h(p)}\\ h\left(p\right)=-\left[p\log_{2}p+\left(1-p\right)\log_{2}\left(1-p\right)\right] \end{array}\right.$$

where *n* denotes the length of the binary sequence, and c(n) represents the total number of new patterns generated by incrementally parsing the sequence from an empty string via the LZ78 algorithm. The term $\log_{2}n$ refers to the maximum number of distinct patterns available for a binary sequence with length *n*. The normalized LZ complexity $C_{\mathrm{norm}}$ is obtained by dividing the original complexity by h(p), where *p* is the occurrence probability of symbol 1 in the sequence. This normalization eliminates the adverse impact of unbalanced symbol-probability distribution. The evaluation criterion of normalized LZ complexity is that values closer to 1 correspond to stronger structural randomness. A value of $C_{\mathrm{norm}}\approx 1$ indicates that the sequence is highly consistent with a random binary string, whereas $C_{\mathrm{norm}}\approx 0$ suggests a highly compressible and predictable sequence. For finite-length chaotic sequences, slight deviations above unity are commonly observed due to finite-size statistical effects.

As depicted in Figure 5d, the normalized Lempel–Ziv complexities of STLEM, sine map, tent map, and logistic map are compared quantitatively, yielding values of 1.1002, 1.0266, 1.1023 and 1.1052, respectively. The sine-map-generated sequence obtains the value closest to the ideal random benchmark of 1, followed by STLEM. The tent map and logistic map deviate further from 1, with logistic map showing the largest departure. Although STLEM is slightly inferior to the sine map in this metric, its normalized Lempel–Ziv complexity still resides within the high-randomness region near 1.

Ideal sequences generated by chaotic maps possess near-zero autocorrelation coefficients, implying negligible linear correlation between sequence elements at different delay positions. This property is essential for image encryption. Natural images exhibit strong spatial correlation, where adjacent pixels have prominent statistical dependence. If the pseudo-random sequences adopted in encryption retain obvious autocorrelation, the resulting ciphertext will preserve residual statistical features of the plaintext. In such cases, adversaries may recover partial plaintext information through statistical analysis such as autocorrelation-based attacks.

Therefore, chaotic sequences with extremely low autocorrelation employed as keystreams and scrambling factors can thoroughly break the inherent spatial correlation of image pixels. This makes ciphertext statistically similar to white noise, effectively defending against correlation-based known-plaintext and chosen-plaintext attacks and guaranteeing the overall security of the encryption scheme. For a discrete chaotic sequence $X=\{x_{1},x_{2},\ldots ,x_{N}\}$ with length *N*, the autocorrelation coefficient at lag *k* is defined as:

(16) $$r\left(k\right)=\frac{\sum_{i=1}^{N-k}\left(x_{i}-\mu \right)\left(x_{i+k}-\mu \right)}{\sum_{i=1}^{N}{\left(x_{i}-\mu \right)}^{2}}$$

where $\mu =\frac{1}{N}\sum_{i=1}^{N}x_{i}$ denotes the mean value of the sequence X, *k* is the lag index (k=0,1,2,…,K, where *K* is the maximum lag considered), and r(k)∈[−1,1] with r(0)=1 by definition. Figure 5e presents the autocorrelation-function curves of all involved chaotic maps. For all four maps, the autocorrelation coefficients rapidly decay to near zero when the lag exceeds zero and fluctuate slightly around the zero baseline without obvious periodic oscillations. This feature indicates that adjacent samples of the STLEM sequence have negligible linear correlation, satisfying fundamental randomness requirements for cryptographic usage.

Overall, the proposed STLEM exhibits excellent chaotic dynamical behavior. In terms of critical quantitative metrics including the Lyapunov exponent, permutation entropy, and approximate entropy, STLEM achieves remarkably higher values than the sine map, tent map and logistic map, which indicates stronger sensitivity to initial values, higher disorder and weaker predictability of the generated time series. In terms of normalized Lempel–Ziv complexity, the sine-map-generated sequence yields the value closest to the ideal random benchmark of unity. The normalized Lempel–Ziv complexity of STLEM is superior to the tent map and logistic map while being marginally higher in complexity than that of the sine map. Nevertheless, all these metrics are concentrated in the high-randomness region near 1, which demonstrates comparable favorable structural randomness for these sequences. Moreover, autocorrelation analysis demonstrates that the sequences produced by STLEM have negligible linear autocorrelation. Although STLEM does not achieve the optimal normalized Lempel–Ziv complexity, it achieves optimal comprehensive performance across all evaluation criteria. The experimental results verify that STLEM possesses richer dynamical characteristics than conventional one-dimensional chaotic maps, and it is well-suited for constructing pseudo-random number generators and chaotic encryption schemes.

### 2.6. NIST SP 800-22 Test

The NIST SP 800-22 [35] test suite serves as the authoritative benchmark for evaluating the randomness of random number generators (RNGs) and the security performance of encryption algorithms. This standard mandates multi-group sequence testing and adopts two core metrics, namely the p-value and pass rate, to quantitatively assess sequence randomness. In accordance with official specifications, the default significance level is set to α=0.01, and the minimum qualified pass rate is defined as 96%.

To ensure fully reproducible randomness verification, this paper explicitly reports all experimental configurations for NIST testing. For NIST input preparation, each retained real-valued chaotic state $x_{n}\in \left[0,1\right)$ was converted into standard binary bitstreams via quantitative sampling:

(17) $$q_{n}=\left\lfloor 2^{32}x_{n}\right\rfloor ,\;b_{n}=\mathrm{bitget}\left(\mathrm{uint}64(q_{n}),1\right),$$

where $b_{n}$ denotes the least-significant bit extracted from 32-bit fixed-point quantization. All generated bits were continuously saved as ASCII characters without separators, which strictly standardizes the bit generation procedure for NIST SP 800-22 testing.

To evaluate the statistical randomness of the proposed STLEM map, 100 independent chaotic sequences were generated under fixed chaotic parameters selected from valid chaotic parameter grids: [7.5,0.3,0.2,−0.5]. All simulations adopted IEEE-754 double-precision floating-point arithmetic. One hundred initial values were uniformly sampled within the range [0.05,0.95). The MATLAB random-number generator was initialized with a fixed seed of 20260614 using the twister algorithm to guarantee reproducibility.

For each initial condition, the first 5000 transient iterative values were discarded to eliminate boundary and initial-state interference. The subsequent $10^{6}$ valid iterations were used to generate one binary bit per iteration according to Equation (17). Consequently, the final test file contained 100 independent bitstreams, each consisting of $10^{6}$ bits. Experiment logging records show that the boundary-correction counter remained zero for all 100 test streams, meaning the boundary-correction branch was never triggered during NIST sampling under this set of parameters and initial-value range. The boundary-correction logic of STLEM was fully enabled in code without any modification or disablement.

Table 2 summarizes the NIST SP-800-22 test results of the STLEM-generated pseudo-random sequences. All uniformity *p*-values are greater than 0.01, and the passing proportion of each test satisfies the NIST statistical threshold. Notably, the Random-Excursions- related tests naturally produce 58 valid subsequences rather than 100 streams, which is an inherent mechanism of the NIST test suite. The proposed STLEM sequence passes all 15 items of the NIST SP 800-22 tests, demonstrating reliable statistical randomness that meets the basic statistical randomness requirements for cryptographic-related usage.

## 3. DNA Encoding Technology

DNA is a linear biopolymer that stores genetic information using four nucleobases: adenine (A), thymine (T), cytosine (C), and guanine (G) [25]. In natural DNA, A and T form a complementary pair, as do C and G. A DNA-inspired representation can encode each 2-bit binary group using one DNA base because the four binary pairs, 00, 01, 10, and 11, correspond to the four DNA bases. To preserve Watson–Crick complementarity, the binary complements 00↔11 and 01↔10 are assigned to the base pairs A–T and C–G, respectively. This constraint gives rise to eight valid DNA coding rules [36], as listed in Table 3.

For an 8-bit grayscale pixel, the binary representation is partitioned from the most significant bit to the least significant bit into four 2-bit groups. Each group is then converted into a DNA base according to a selected coding rule. For example, the pixel value 182 is represented as ${\left[10110110\right]}_{2}$ and partitioned into (10,11,01,10). Under Rule 1, the corresponding DNA sequence is GTCG; under Rule 2, it is CTGC. The binary representation is not altered during this encoding step; instead, different coding rules produce different DNA-base representations of the same pixel value. The original pixel value is recovered by applying the inverse mapping associated with the same coding rule.

For subsequent DNA-domain operations, the DNA bases are assigned integer labels as

(18) ϕ(A)=0,ϕ(G)=1,ϕ(C)=2,ϕ(T)=3.

Given two DNA bases *X* and *Y*, the five operations employed in this work are defined under modulo-4 arithmetic as

(19) $$\begin{array}{cc} O_{1}\left(X,Y\right) & =\phi^{-1}\left((\phi (X)+\phi (Y))\;\mathrm{mod}\;4\right),\\ O_{2}\left(X,Y\right) & =\phi^{-1}\left((\phi (X)-\phi (Y))\;\mathrm{mod}\;4\right),\\ O_{3}\left(X,Y\right) & =\phi^{-1}\left(\phi (X)⊕\phi (Y)\right),\\ O_{4}\left(X,Y\right) & =\phi^{-1}\left((\phi (X)+\phi (Y)+1)\;\mathrm{mod}\;4\right),\\ O_{5}\left(X,Y\right) & =\phi^{-1}\left((\phi (X)-\phi (Y)-1)\;\mathrm{mod}\;4\right). \end{array}$$

Here, ⊕ denotes bitwise exclusive OR (XOR). Operations $O_{4}$ and $O_{5}$ are cyclically shifted variants of conventional DNA addition and subtraction, respectively. The positive cyclic direction is defined as A→G→C→T→A. Accordingly, the constant +1 in $O_{4}$ induces a one-step positive shift, whereas the constant −1 in $O_{5}$ induces a one-step negative shift. DNA arithmetic operations are as shown in the Table 4.

These DNA-domain coding rules and arithmetic operations are key-selectable and reversible, which enables them to be embedded within a permutation–diffusion encryption architecture.

Numerous existing DNA-based image encryption approaches employ a single fixed global DNA coding rule for the entire image, where all pixels are encoded and decoded according to one predefined mapping. This static mapping strategy may introduce two potential security vulnerabilities. First, under chosen-plaintext attack conditions, the pixel-to-DNA-base mapping stays invariant over the whole image domain. Adversaries can exploit known plaintext-ciphertext pairs to mine statistical biases originating from this static transformation. Second, in the scenario where permutation operations are partially undermined, identical plaintext pixel values at distinct spatial coordinates always produce identical DNA symbol sequences, which risks leaking plaintext frequency information.

To mitigate these risks, this work adopts an adaptive position-dependent encoding strategy, in which the coding rule for each pixel block is dynamically determined by real-time outputs from the STLEM chaotic map such that distinct DNA mapping rules are assigned to different spatial positions. This design brings three key benefits. It enables mapping diversification, whereby identical pixel values appearing at different spatial positions are converted into distinct DNA symbol strings, suppressing plaintext-frequency leakage even when parts of the permutation layer become compromised. It also provides key-dependent variability: as coding rules stem from secret chaotic sequences, tiny perturbations applied to the master key or chaotic parameters will fully reshuffle the sequence of coding rules, increasing the computational burden for adversaries seeking to build static mapping tables linking pixel intensities and DNA symbols. Furthermore, the scheme supports seamless reversibility, since the full rule sequence can be faithfully reconstructed on the decryption side using matching keys and chaotic parameters, with no extra metadata describing coding rules requiring transmission or storage and therefore incurring zero additional metadata overhead.

It should be emphasized that adaptive position-dependent encoding cannot substitute permutation-diffusion mechanisms. Instead, it serves as an auxiliary confusion layer that cooperates with chaotic permutation and DNA-domain chained diffusion. The security improvement brought by this adaptive mechanism relies heavily on the statistical randomness of underlying chaotic sequences. For the proposed STLEM map, such randomness has been verified through NIST SP 800-22 tests and multiple dynamical analyses.

## 4. Proposed Encryption Scheme

**Figure 6:**
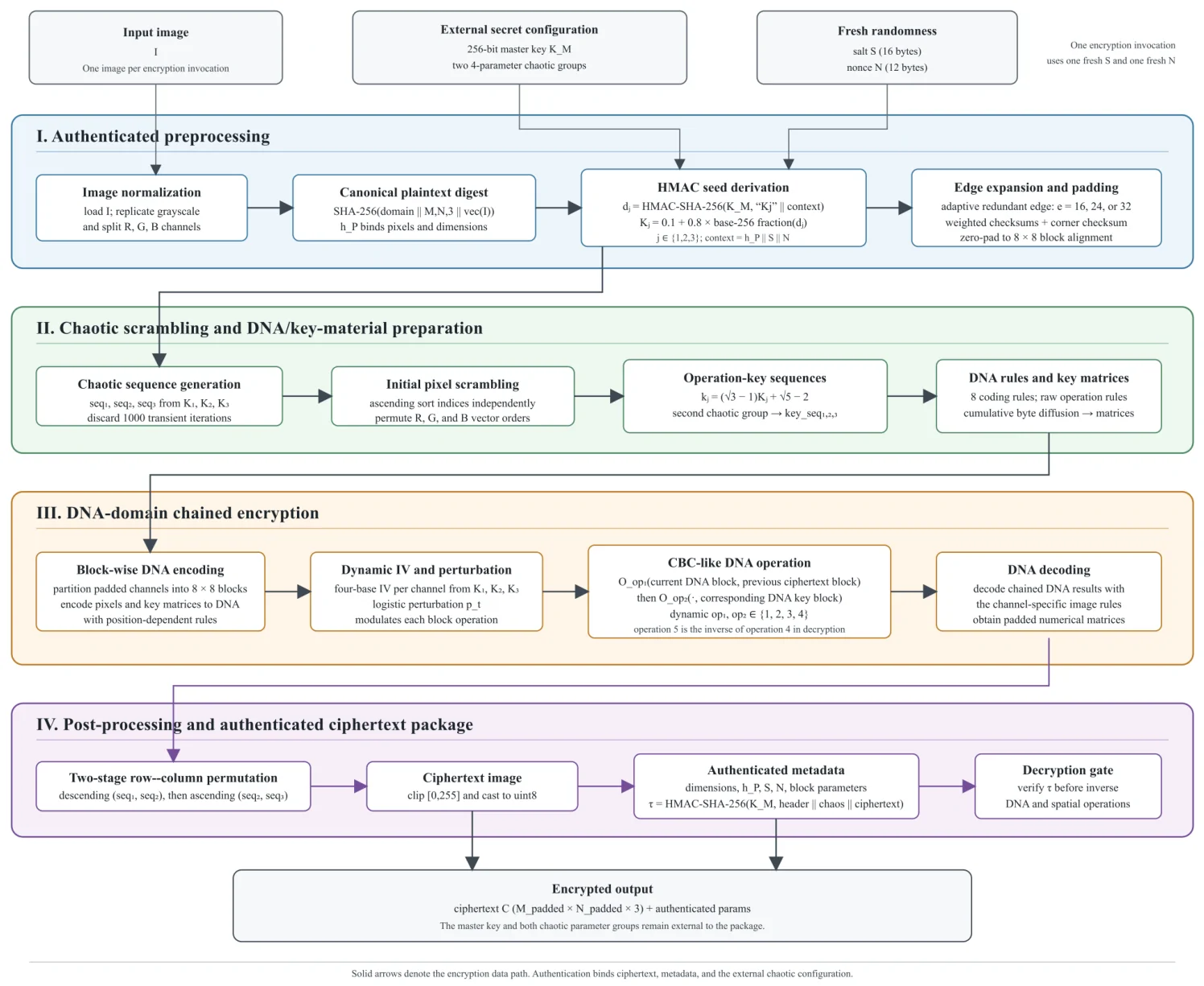
Encryption Workflow.

The encryption function operates on one input image I per invocation. A 256-bit master key $K_{M}$ and two four-parameter chaotic groups are supplied externally at run time. They are not included in the stored ciphertext metadata. Figure 6 summarizes the image encryption and authenticated-output procedure.

### 4.1. Preprocessing and Authenticated Chaotic-Seed Derivation

**Step 1: Image loading and channel decomposition.** The input image is read as an 8-bit array. If the input is grayscale, its single channel is replicated to form a three-channel representation. After this normalization, let $I\in {\left\{0,\ldots ,255\right\}}^{M\times N\times 3}$ denote the input image. The three channels are decomposed into the red, green, and blue matrices R, G, and B, respectively.

**Step 2: Canonical plaintext hashing and seed derivation.** The implementation hashes a canonical byte representation rather than a decimal character string. Specifically,

(20) $$h_{P}=\mathrm{SHA}\text{-}256\left(\mathrm{DNA}\text{-}\mathrm{IMAGE}\text{-}\mathrm{PLAINTEXT}\text{-}V1\;\|\;{\mathrm{uint}32}_{\mathrm{BE}}\left(M,N,3\right)\;\|\;{\mathrm{vec}}_{\mathrm{MATLAB}}\left(I\right)\right),$$

where ‖ denotes byte concatenation and ${\mathrm{vec}}_{\mathrm{MATLAB}}\left(\;\cdot \;\right)$ denotes MATLAB column-major serialization. Hence, the dimensions and all RGB pixels contribute to the 256-bit plaintext digest.

For each encryption invocation, a 16-byte salt s and a 12-byte nonce n are drawn from java.security.SecureRandom. The seed-derivation context is

(21) $$c=\mathrm{DNA}\text{-}\mathrm{CHAOS}\text{-}\mathrm{SEED}\text{-}V1\;\|\;h_{P}\;\|\;s\;\|\;n.$$

Three independent HMAC outputs are calculated from the 256-bit master key:

(22) $$d_{j}=\mathrm{HMAC}\text{-}\mathrm{SHA}\text{-}256\left(K_{M},K\;j\;\|\;c\right),\;j\in \left\{1,2,3\right\}.$$

The first eight bytes of each $d_{j}$ are interpreted as a base-256 fraction and mapped to the chaotic operating interval:

(23) $$z_{j}=\sum_{q=1}^{8}\frac{d_{j}\left(q\right)}{256^{q}},\;K_{j}=0.1+0.8\;z_{j},\;j\in \left\{1,2,3\right\}.$$

Thus, $K_{1}$, $K_{2}$, and $K_{3}$ are real-valued chaotic seeds rather than three independent 256-bit encryption keys. The authenticated chaotic-seed derivation procedure is described in Algorithm 1.
**Algorithm 1** Authenticated Chaotic-Seed Derivation**Require:** Image I and master key $K_{M}$**Ensure:** $K_{1},K_{2},K_{3},h_{P},s,n$  1:Replicate I to three channels if it is grayscale  2:$h_{P}\leftarrow \mathrm{SHA}\text{-}256(\mathrm{DNA}\text{-}\mathrm{IMAGE}\text{-}\mathrm{PLAINTEXT}\text{-}V1\;\|\;{\mathrm{uint}32}_{\mathrm{BE}}\left(M,N,3\right)\;\|\;{\mathrm{vec}}_{\mathrm{MATLAB}}\left(I\right))$  3:s←SecureRandom(16); n←SecureRandom(12)  4:$c\leftarrow \mathrm{DNA}\text{-}\mathrm{CHAOS}\text{-}\mathrm{SEED}\text{-}V1\;\|\;h_{P}\;\|\;s\;\|\;n$  5:**for** j=1 **to** 3 **do**  6:    $d_{j}\leftarrow \mathrm{HMAC}-\mathrm{SHA}-256(K_{M},K$j‖ c)  7:    $z_{j}\leftarrow \sum_{q=1}^{8}d_{j}\left(q\right)/256^{q}$  8:    $K_{j}\leftarrow 0.1+0.8\;z_{j}$  9:**end for**

**Step 3: Adaptive redundant-edge construction.** For each channel X∈{R,G,B}, the edge width is selected according to

(24) $$e=\left\{\begin{array}{cc} 16, & MN<256^{2},\\ 24, & 256^{2}\leq MN<512^{2},\\ 32, & MN\geq 512^{2}. \end{array}\right.$$

An all-zero carrier of dimensions (M+2e)×(N+2e) is initialized, and X is embedded in its central region. The first and last min(4,e) border lines are filled with weighted row-wise and column-wise checksums modulo 256. The four corner regions are filled with

(25) $$v_{\mathrm{corner}}=\;\mathrm{mod}\;\left(\sum_{m=1}^{M}\sum_{n=1}^{N}X\left(m,n\right),256\right).$$

**Step 4: Block-aligned zero padding.** With block size b=8, the expanded matrices are zero-padded only on their bottom and right sides to obtain

(26) $$M_{\mathrm{padded}}=b\left\lceil \frac{M+2e}{b}\right\rceil ,\;N_{\mathrm{padded}}=b\left\lceil \frac{N+2e}{b}\right\rceil .$$

The padded channel matrices can consequently be divided into non-overlapping 8×8 blocks.

### 4.2. Chaotic Scrambling and Key Preparation

**Step 5: Chaotic sequences and initial scrambling.** Starting from $K_{1}$, $K_{2}$, and $K_{3}$, three sequences ${\mathrm{seq}}_{1}$, ${\mathrm{seq}}_{2}$, and ${\mathrm{seq}}_{3}$ of length $L=M_{\mathrm{padded}}N_{\mathrm{padded}}$ are generated after discarding 1000 transient iterations. This implementation adopts the proposed chaotic mapping STLEM. The parameter group (a,b,c,d) is supplied externally. Values smaller than $10^{-12}$ and larger than $1-10^{-12}$ are replaced by 0.48 and 0.52, respectively. The ascending-sort indices of ${\mathrm{seq}}_{1}$, ${\mathrm{seq}}_{2}$, and ${\mathrm{seq}}_{3}$ independently permute the vectorized R, G, and B matrices.

**Step 6: Operation-key sequences, DNA rules, and key matrices.** The second external chaotic group is initialized with

(27) $$\begin{array}{cc} k_{1} & =\left(\sqrt{3}-1\right)\;K_{1}+\sqrt{5}-2,\\ k_{2} & =\left(\sqrt{3}-1\right)\;K_{2}+\sqrt{5}-2,\\ k_{3} & =\left(\sqrt{3}-1\right)\;K_{3}+\sqrt{5}-2. \end{array}$$

This produces ${\mathrm{kseq}}_{1}$, ${\mathrm{kseq}}_{2}$, and ${\mathrm{kseq}}_{3}$. With dna_step=8 and T=⌊L/8⌋, the image-DNA rule sequences are

(28) $$\begin{array}{cc} r_{R} & =\;\mathrm{mod}\;(\left\lfloor 100*{\mathrm{seq}}_{1}\left(1:T\right)\right\rfloor ,8)+1,\\ r_{G} & =\;\mathrm{mod}\;(\left\lfloor 100*{\mathrm{seq}}_{2}\left(1:T\right)\right\rfloor ,8)+1,\\ r_{B} & =\;\mathrm{mod}\;(\left\lfloor 100*{\mathrm{seq}}_{3}\left(1:T\right)\right\rfloor ,8)+1. \end{array}$$

The three key-DNA rule sequences are constructed from ${\mathrm{kseq}}_{1}$, ${\mathrm{kseq}}_{2}$, and ${\mathrm{kseq}}_{3}$ in the same manner. Two raw operation-rule sequences are generated from ${\mathrm{kseq}}_{1}$ and ${\mathrm{kseq}}_{2}$ by reduction modulo five.

The byte sequences for the key matrices are initialized by

(29) $$u_{j}=\left\lfloor 256*{\mathrm{kseq}}_{j}\left(1:L\right)\right\rfloor ,\;j\in \left\{1,2,3\right\},$$

and diffused according to

(30) $$u_{j}\left(i\right)=\;\mathrm{mod}\;\left(u_{j}\left(i\right)+u_{j}\left(i-1\right),256\right),\;i=2,\ldots ,L.$$

Each diffused sequence is then reshaped into an $M_{\mathrm{padded}}\times N_{\mathrm{padded}}$ key matrix.

### 4.3. DNA-Domain Chained Encryption

**Step 7: Position-dependent DNA encoding.** Each padded color channel and its corresponding key matrix are partitioned into non-overlapping 8×8 blocks. Every 8-bit value is divided into four two-bit groups and encoded as a four-symbol DNA sequence. One of the eight admissible DNA coding rules is selected from the position-dependent rule sequence. The RGB channels adopt $r_{R}$, $r_{G}$, and $r_{B}$, whereas each key matrix uses its corresponding key-DNA rule sequence.

**Step 8: Dynamic-IV CBC-like DNA operation.** For X∈{R,G,B}, a four-base initial vector ${\mathrm{IV}}_{X}$ is regenerated from $K_{X}$. A logistic perturbation sequence is formed as

(31) $$p_{0}=\mathrm{mod}\left(K_{1}+K_{2},1\right),\;\rho =3.9+0.1\;K_{3},\;p_{t}=\rho \;p_{t-1}\left(1-p_{t-1}\right).$$

For block *t*, the two dynamic operation indices are

(32) $$o_{1}=\mathrm{mod}\left(q_{1}\left(t\right)+\left\lfloor 4p_{t}\right\rfloor ,4\right)+1,\;o_{2}=\mathrm{mod}\left(q_{2}\left(t\right)+\left\lfloor 3p_{t}\right\rfloor ,4\right)+1.$$

The ciphertext DNA block is computed in two stages:

(33) $$C_{X,t}^{\mathrm{DNA}}=O_{o_{2}}\left(O_{o_{1}}\left(P_{X,t}^{\mathrm{DNA}},C_{X,t-1}^{\mathrm{DNA}}\right),K_{X,t}^{\mathrm{DNA}}\right).$$

The first chained input is the repeated block-sized representation of ${\mathrm{IV}}_{X}$, and each subsequent chained input is the preceding ciphertext block. Although the helper function defines five DNA operations, the modulo-four update in Equation (32) selects operations 1–4 on the encryption path. Operation 5 is adopted by the inverse routine when reversing operation 4. The generated DNA strings are then decoded with the corresponding image-DNA rules.

### 4.4. Post-Processing, Authentication, and Decryption

**Step 9: Two-stage spatial permutation and ciphertext generation.** For every decoded color channel, the first spatial permutation applies descending row-sort and column-sort indices from ${\mathrm{seq}}_{1}$ and ${\mathrm{seq}}_{2}$, respectively. The second permutation applies ascending row-sort and column-sort indices from ${\mathrm{seq}}_{2}$ and ${\mathrm{seq}}_{3}$, respectively. The resulting values are clipped to [0,255], cast to uint8, and concatenated to form the RGB ciphertext image. The overall encryption process is as shown in the Algorithm 2.
**Algorithm 2** Overall Encryption Pipeline**Require:** Input RGB image I, 256-bit master key $K_{M}$, two chaos-parameter groups $C_{1},C_{2}$**Ensure:** ciphertext image C, authenticated metadata params  1:**Preprocessing and seed derivation**  2:Decompose I into R,G,B; replicate channels if grayscale  3:Compute plaintext hash $h_{P}$, generate random s (16-byte), n (12-byte)  4:Compute chaotic seeds $K_{1},K_{2},K_{3}$ via Algorithm 1  5:**Dynamic redundant-edge construction and block padding**  6:Compute edge width *e* from M×N size according to Equation (24)  7:Perform edge expansion with weighted checksums and corner checksum Equation (25)  8:Zero-pad to 8×8 block aligned dimensions $M_{\mathrm{padded}},N_{\mathrm{padded}}$  9:**Chaotic scrambling and key preparation**10:Generate ${\mathrm{seq}}_{1},{\mathrm{seq}}_{2},{\mathrm{seq}}_{3}$ using STLEM, discard $T_{0}=1000$ transient iterations11:Initial pixel-vector permutation using ascending sort indices of chaotic sequences12:Compute $k_{1},k_{2},k_{3}$ from Equation (27)13:Generate key sequences ${\mathrm{kseq}}_{1},{\mathrm{kseq}}_{2},{\mathrm{kseq}}_{3}$14:Compute position-dependent DNA rule sequences $r_{R},r_{G},r_{B}$ and key-DNA rules15:Build diffused key matrices via Equations (29) and (30)16:**DNA-domain chained encryption**17:Partition padded channels and key matrices into non-overlapping 8×8 blocks18:Encode each pixel block into DNA sequences with position-dependent coding rules19:Generate logistic perturbation sequence $\left\{p_{t}\right\}$ Equation (31)20:Initialize block-size DNA initial vectors ${\mathrm{IV}}_{R},{\mathrm{IV}}_{G},{\mathrm{IV}}_{B}$21:**for** each block index t=1 total_blocks **do**22:   compute dynamic operation indices $o_{1},o_{2}$ Equation (32)23:   chained DNA CBC-like update Equation (33)24:**end for**25:DNA decode blocks back to numerical pixel matrices26:**Two-stage spatial permutation**27:First permutation: descending row-column sort indices of ${\mathrm{seq}}_{1},{\mathrm{seq}}_{2}$28:Second permutation: ascending row-column sort indices of ${\mathrm{seq}}_{2},{\mathrm{seq}}_{3}$29:Clip values to [0,255], cast to uint8 to obtain ciphertext C30:**Authentication metadata generation**31:Fill metadata structure params with dimensions, padding, hash, salt, nonce etc.32:Compute HMAC-SHA-256 authentication tag τ Equation (34)33:params.auth_tag←τ34:**return** C,params

**Step 10: Authenticated metadata and inverse recovery.** The implementation stores the version identifier, $h_{P}$, s, n, dimensions, padding and block parameters, and transient length in params. These values, the ciphertext, and the binary representation of both external chaotic parameter groups are authenticated by

(34) $$\tau =\mathrm{HMAC}\text{-}\mathrm{SHA}\text{-}256\left(K_{M},\mathrm{DNA}\text{-}\mathrm{IMAGE}\text{-}\mathrm{AUTH}\text{-}V1\;\|\;h_{\mathrm{meta}}\;\|\;g_{\mathrm{chaos}}\;\|\;{\mathrm{vec}}_{\mathrm{MATLAB}}\left(C\right)\right).$$

The tag τ is verified prior to decryption. The receiver then regenerates the chaotic seeds, IVs, sequences, and key matrices; inverts the two spatial permutations and DNA operations; reverts the initial-vector permutations; and removes the redundant edge. A modified ciphertext, modified metadata, or an incorrect external configuration triggers authentication failure before the inverse transformations are performed. The decryption process is as shown in the Algorithm 3.
**Algorithm 3** Authenticated Decryption Pipeline**Require:** ciphertext image C, metadata params, master key $K_{M}$, chaos groups $C_{1},C_{2}$**Ensure:** recovered original RGB image $I_{\mathrm{out}}$  1:**Authentication verification**  2:Recompute authentication tag from input ciphertext and metadata  3:**if** recomputed tag ≠params.auth_tag **then**  4:    **ERROR**: Authentication failure; abort decryption  5:**end if**  6:Regenerate $h_{P},s,n$ from metadata  7:Re-derive chaotic seeds $K_{1},K_{2},K_{3}$ by Algorithm 1  8:Regenerate all STLEM chaotic sequences ${\mathrm{seq}}_{1},{\mathrm{seq}}_{2},{\mathrm{seq}}_{3}$, key sequences ${\mathrm{kseq}}_{1,2,3}$  9:Reconstruct DNA rule sequences, key matrices, perturbation sequence $\left\{p_{t}\right\}$10:**Inverse two-stage row-column permutation**11:Apply inverse sorting operations to revert second-stage then first-stage spatial permutation12:Partition ciphertext and key matrices to 8×8 blocks13:Encode ciphertext blocks and key-matrix blocks into DNA representations14:Recover DNA initial vectors ${\mathrm{IV}}_{R},{\mathrm{IV}}_{G},{\mathrm{IV}}_{B}$ from $K_{1,2,3}$15:**for** t=1 total_blocks **do**16:   get dynamic $o_{1},o_{2}$; fetch inverse-operation indices via Supplementary Algorithm S417:   perform inverse chained DNA CBC-like decryption18:**end for**19:DNA decode blocks back to numerical padded matrices20:Apply inverse initial pixel-vector permutation21:Remove redundant edge region, crop to original M×N dimensions22:Clip pixel values [0,255], cast to uint823:**return** $I_{\mathrm{out}}$

Detailed low-level sub-routines including STLEM iteration, DNA encoding/decoding, DNA arithmetic operations and inverse-operation lookup are provided in Supplementary Material Algorithms S1–S4.

## 5. Simulation Results and Security Analysis

**Figure 7:**
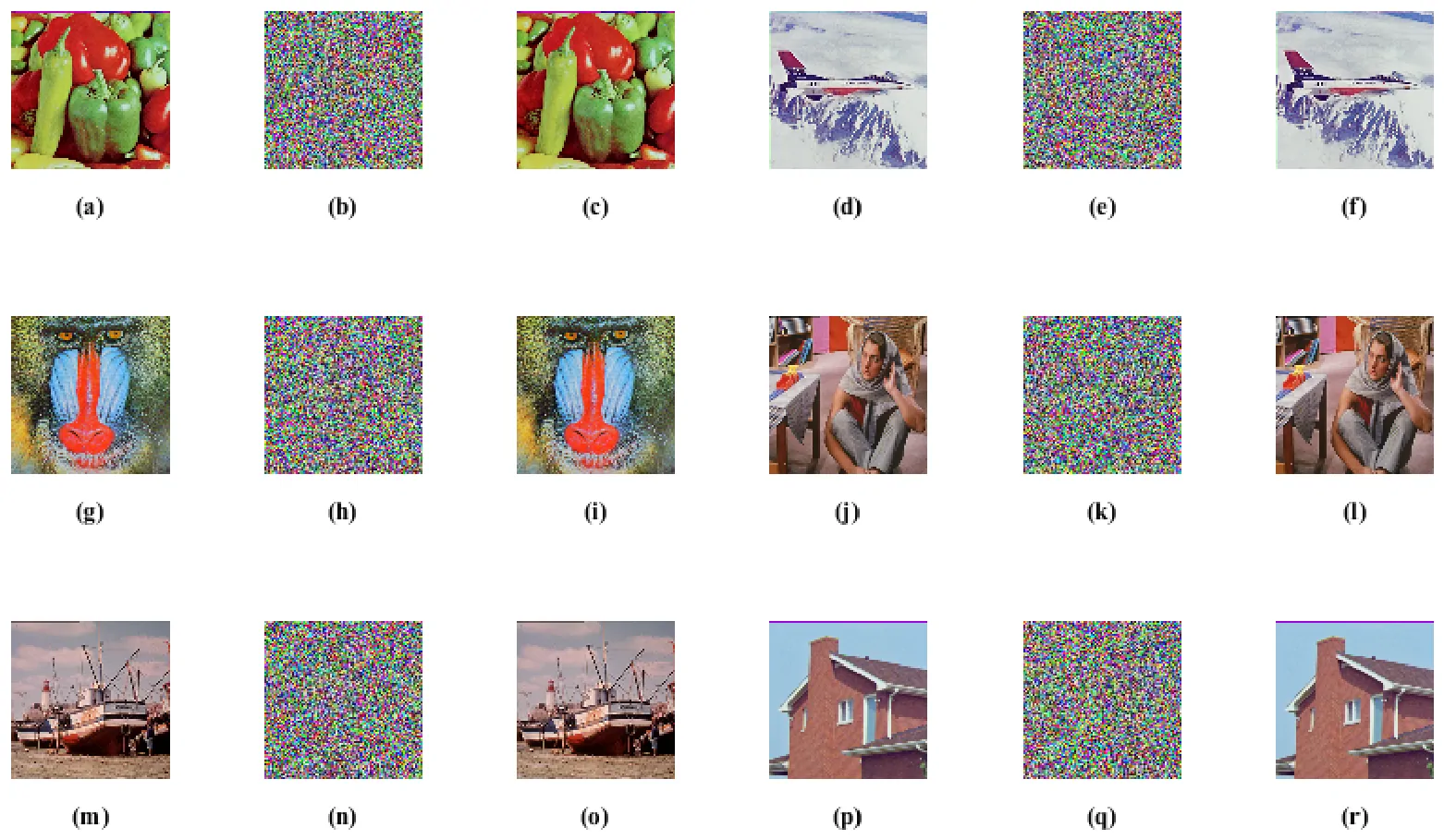
Encryption and decryption results for multiple standard test images. (**a**,**d**,**g**,**j**,**m**,**p**) Plain images of Airplane, Baboon, Lena, Boat, House, and Woman; (**b**,**e**,**h**,**k**,**n**,**q**) Encrypted ciphertext images; (**c**,**f**,**i**,**l**,**o**,**r**) Decrypted images.

The proposed algorithm was implemented in MATLAB R2020a and evaluated using standard color images of size 256×256 and 512×512, including *Peppers*, *Airplane*, *Baboon*, *Boats*, *Barbara*, and *House*. Figure 7 presents representative plaintext, ciphertext, and correctly decrypted images. The cipher images exhibit a noise-like appearance, whereas the plaintext images are recovered exactly with valid secret inputs.

### 5.1. Simulation Settings and Authentication Policy

All numerical simulations, security tests, and performance evaluations are implemented in MATLAB R2020a. To guarantee full reproducibility of all experimental results presented in this manuscript, we explicitly report the fixed secret parameters adopted throughout our tests. The 256-bit master key is given as the 64-character hexadecimal string 83036DC51D99A0F48F7E13566111B7ECE888CFD7555FE7E32C129FAFE419BB3C. For the chaos-parameter groups, we assign $C_{1}=\left[a_{1},b_{1},c_{1},d_{1}\right]$ and $C_{2}=\left[a_{2},b_{2},c_{2},d_{2}\right]$ with $\left[a_{1},b_{1},c_{1},d_{1}\right]=\left[1.943434114040,\;1.058068757598,\;-0.016468220770,\;-1.145415570222\right]$ and $\left[a_{2},b_{2},c_{2},d_{2}\right]=\left[1.294846566143,\;1.609261352101,\;-0.047833817582,\;-0.297466354660\right]$, respectively. All selected chaos parameters lie within the revised admissible intervals a∈[0,9], b∈[−1.6,2], c∈[−0.5,0.5], and d∈[−2,0].

All chaotic iterations use IEEE-754 double-precision floating-point arithmetic. A fixed transient discard length $T_{0}=1000$ iterations is applied before collecting chaotic output samples. Standard test color images Peppers, Airplane, Baboon, Boat, Barbara and House with resolution 256×256 and 512×512 are adopted for evaluation.

Authentication policy note. Under normal decryption workflow, HMAC-SHA-256 tag verification is always executed before any decryption operation. Ciphertext tampering, noise corruption, cropping-damaged ciphertext or wrong secret inputs will trigger authentication failure and reject decryption directly. For noise-attack, cropping-attack and hybrid-attack experiments in this paper, the authentication check is *manually bypassed only for observing error-propagation characteristics*. This bypass is only for simulation analysis purposes and does not represent the secure default behavior of the cryptosystem.

Unless otherwise stated, HMAC-SHA-256 verification is performed before decryption. Thus, incorrect secret inputs or modified ciphertexts cause authentication failure and rejection in normal operation. Authentication is bypassed only in four diagnostic tests: (i) wrong-key or wrong-parameter visualization within the key-sensitivity test; (ii) cropping-corruption analysis; (iii) noise-corruption analysis; (iv) hybrid-tampering analysis. The bypassed decryptions are used solely to visualize sensitivity and error-propagation behavior and do not represent normal operation.

In the key-sensitivity test, decryption using the nominal secret inputs and decryption using each perturbed configuration with its corresponding newly generated ciphertext are performed with authentication verification enabled. The bypass applies only when the nominal ciphertext is deliberately decrypted using an incorrect master key or perturbed chaos parameter. Similarly, the cropping, noise, and hybrid-tampering experiments bypass authentication only to quantify the error-propagation behavior of the underlying image-decryption procedure. Under normal operation, all such altered ciphertexts are rejected before decryption.

The key-space analysis does not involve ciphertext decryption. The chosen-plaintext test decrypts each newly generated ciphertext using its valid ciphertext–tag pair with authentication enabled. Histogram, adjacent-pixel correlation, and information-entropy analyses operate directly on ciphertext data. For the differential-attack test, one plaintext sample is modified and the resulting valid ciphertexts are compared using NPCR and UACI; therefore, authentication verification is not bypassed.

### 5.2. Key Space

The secret configuration of the proposed encryption scheme consists of a 256-bit master key $K_{M}$ and two independent chaos-parameter groups,

(35) $$K_{\mathrm{cfg}}=\left(K_{M},C_{1},C_{2}\right),$$

where

(36) $$C_{i}=\left(a_{i},b_{i},c_{i},d_{i}\right),\;i\in \left\{1,2\right\}.$$

For both chaos-parameter groups, the admissible intervals are defined as

(37) a∈[0,9],b∈[−1.6,2],c∈[−0.5,0.5],d∈[−2,0].

A finite numerical key-space estimate requires an explicit parameter quantization rule. Here, each chaos parameter is assumed to be selected independently from its admissible interval with resolution $\varepsilon =10^{-15}$. Therefore, the numbers of selectable values are

(38) $$\begin{array}{cccc} N_{a} & =\left\lfloor \frac{9-0}{\varepsilon }\right\rfloor +1, & N_{b} & =\left\lfloor \frac{2-(-1.6)}{\varepsilon }\right\rfloor +1,\\ N_{c} & =\left\lfloor \frac{0.5-(-0.5)}{\varepsilon }\right\rfloor +1, & N_{d} & =\left\lfloor \frac{0-(-2)}{\varepsilon }\right\rfloor +1. \end{array}$$

Substituting the interval lengths:

(39) $$\begin{array}{cccc} N_{a} & =\left\lfloor \frac{9}{\varepsilon }\right\rfloor +1\approx 9.0\times 10^{15}, & N_{b} & =\left\lfloor \frac{3.6}{\varepsilon }\right\rfloor +1\approx 3.6\times 10^{15},\\ N_{c} & =\left\lfloor \frac{1.0}{\varepsilon }\right\rfloor +1\approx 1.0\times 10^{15}, & N_{d} & =\left\lfloor \frac{2.0}{\varepsilon }\right\rfloor +1\approx 2.0\times 10^{15}. \end{array}$$

Accordingly, the key space contributed by one chaos-parameter group is

(40) $$\begin{array}{ccc} N_{C} & = & N_{a}\;N_{b}\;N_{c}\;N_{d}\\ \; & \approx & \left(9.0\times 10^{15}\right)\;\left(3.6\times 10^{15}\right)\;\left(1.0\times 10^{15}\right)\;\left(2.0\times 10^{15}\right)\\ \; & \approx & 6.48\times 10^{61}. \end{array}$$

Convert $N_{C}$ to base-2 representation:

$$\log_{2}\left(N_{C}\right)\approx \log_{2}\left(6.48\times 10^{61}\right)\approx 204.472.$$

Since two independent chaos-parameter groups are employed, the overall key space is calculated as

(41) $$\begin{array}{ccc} \left|K_{\mathrm{cfg}}\right| & = & 2^{256}{\left(N_{C}\right)}^{2}\\ \; & \approx & 2^{256\;+\;2\times 204.472}\\ \; & = & 2^{664.944}\approx 1.31\times 10^{200}. \end{array}$$

The salt, nonce, plaintext hash, HMAC-SHA-256-derived chaotic initial states, DNA initial vectors, dynamic DNA encoding and operation rules, perturbation sequence, and authentication tag are not counted as independent secret components. These quantities are either public metadata or are deterministically derived from the master key and chaos parameters; therefore, including them would double-count the secret entropy.

Hence, under the stated key-generation and quantization assumptions, the key space is larger than $2^{664}$, conventionally reported as approximately $2^{665}$. This key space is substantially larger than the commonly accepted $2^{128}$ threshold against exhaustive-search attacks.

The above value $2^{665}$ represents the *nominal key space* under ideal infinite-precision arithmetic. In practical MATLAB implementations, all chaotic iterations are performed using 64-bit double-precision floating-point numbers, which provide about 15–16 significant decimal digits. Finite-precision arithmetic introduces two well-known side-effects for chaotic maps: orbit degeneration, periodicity re-appearance, and possible merging of distinct trajectories. Consequently, the *effective key space* is smaller than the nominal theoretical value.

Several countermeasures are embedded within the proposed encryption framework to alleviate finite-precision induced dynamical degradation. Prior to extracting usable chaotic sequences, a transient of 1000 iterations is discarded to suppress numerical artifacts originating from quantized initial conditions. Rather than being directly provided by users, the chaotic initial seeds $K_{1},K_{2},K_{3}$ are deterministically derived through HMAC-SHA-256, combining the 256-bit master key, plaintext hash, salt, and nonce. This derivation mechanism distributes secret entropy uniformly across initial states and prevents the adoption of ill-conditioned starting values that could degrade chaotic behavior. Furthermore, the utilization of two independent chaos-parameter groups for chaotic sequence generation and key-matrix construction raises the computational complexity of partial-key recovery attempts, further strengthening resistance against brute-force cryptanalysis under finite-precision arithmetic.

Even considering the shrinkage caused by finite-precision dynamics, the effective key space of the proposed scheme remains significantly beyond $2^{128}$, which is computationally infeasible for brute-force exhaustive search under current computing capabilities.

### 5.3. Key Sensitivity

A secure encryption system is required to exhibit high key-sensitivity performance. Specifically, a reliable cryptosystem must be extremely sensitive to tiny variations in secret parameters. For the proposed image encryption scheme, key sensitivity means that a slight modification to the secret configuration produces completely different encryption or decryption outputs. This property serves as a crucial criterion for evaluating the security of the designed encryption algorithm. Consistent with most existing related studies, tiny perturbations are introduced to the secret parameters for key-sensitivity testing.

**Figure 8:**
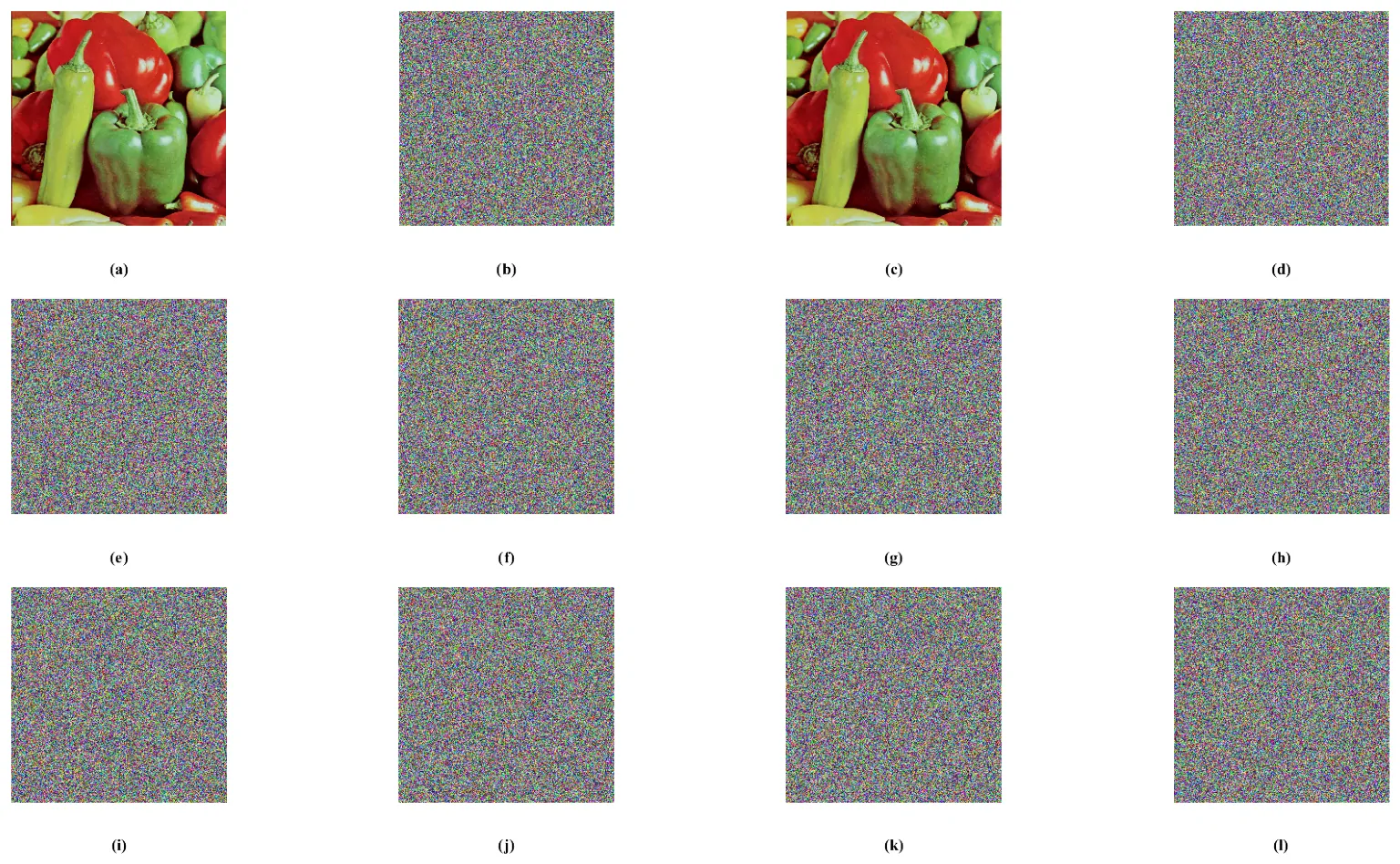
Visual assessment of master-key and chaos-parameter sensitivity of the proposed encryption scheme. (**a**) Original plaintext image; (**b**) ciphertext generated using the nominal secret inputs; (**c**) correctly decrypted image. Panel (**d**) shows the decryption result after flipping the least significant bit of the first byte of the 256-bit master key. Panels (**e**–**h**) show the results obtained by individually perturbing $a_{1}$, $b_{1}$, $c_{1}$, and $d_{1}$, respectively, in the first chaos-parameter group; panels (**i**–**l**) show the corresponding results obtained by perturbing $a_{2}$, $b_{2}$, $c_{2}$, and $d_{2}$ in the second group. Each chaos parameter is perturbed by $\Delta p=\max\left(1,|p|\right)\times 10^{-14}$. For visualization only, authentication verification is bypassed in panels (**d**–**l**).

Figure 8 illustrates the decryption results obtained using the correct secret configuration and slightly perturbed incorrect parameters. The experimental results demonstrate that such minor parameter variations lead to dramatically different decrypted images. Decrypted outputs obtained with perturbed keys are completely unrecognizable and bear no resemblance to the original plaintext image. Therefore, the proposed algorithm possesses excellent key sensitivity, and accurate plaintext recovery via key guessing is infeasible.

### 5.4. Statistic Analysis

#### 5.4.1. Histogram

**Figure 9:**
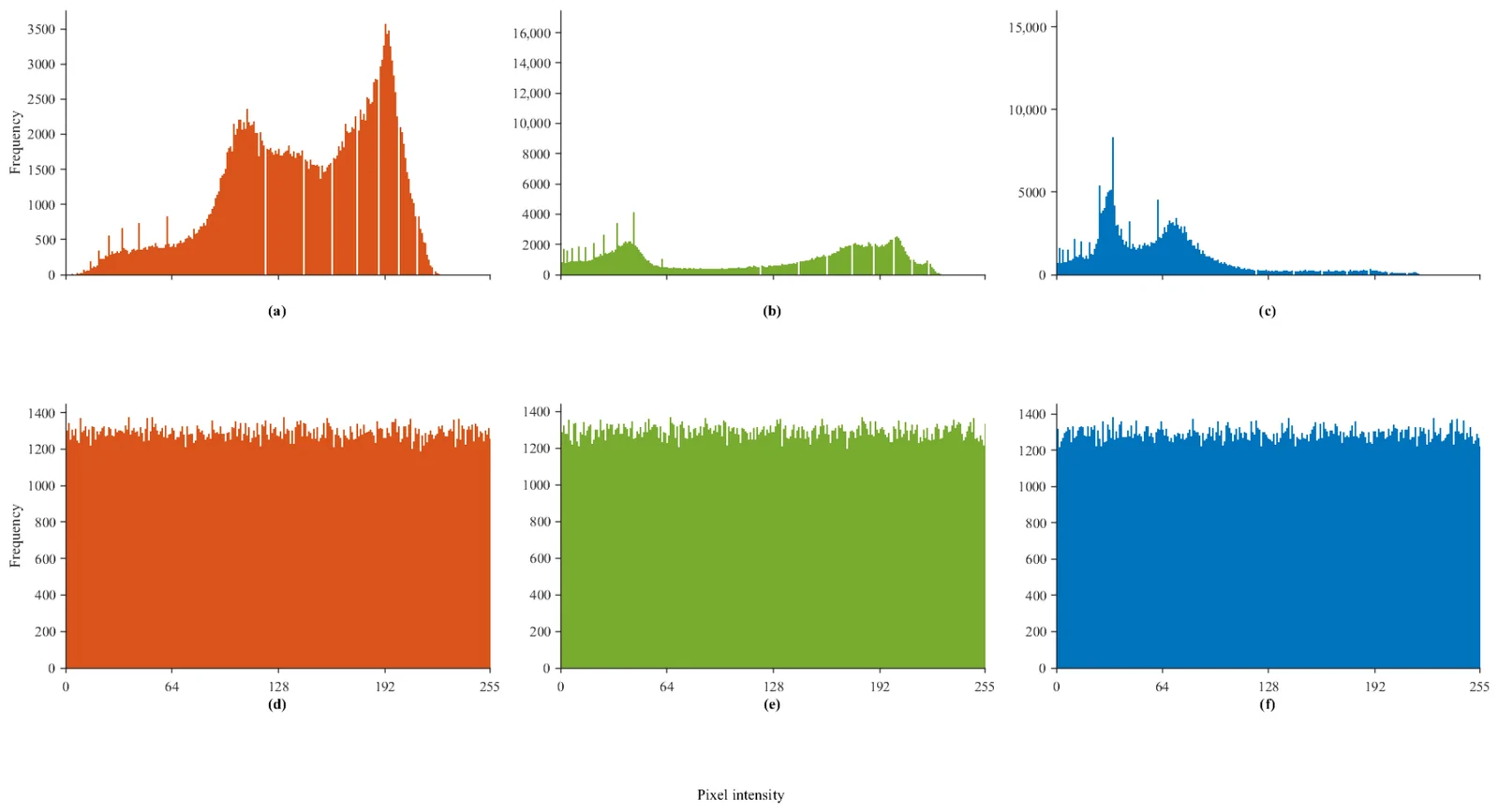
RGB-channel histograms of the original and encrypted images: (**a**–**c**) original image; (**d**–**f**) encrypted image.

Pixel intensity distribution of an image can be intuitively reflected by its histogram. Natural plaintext images typically present irregular and fluctuating histogram distributions with distinct pixel-concentration characteristics, which contain abundant valid image information. After image encryption, the histogram of the cipher image is expected to exhibit a flat and uniform distribution. Such uniform histogram features can effectively resist statistical-histogram attacks, which constitute a vital indicator of excellent encryption performance. Figure 9 compares the histograms of the original *Peppers* image and its corresponding encrypted image. Apparently, the original image possesses a fluctuating and uneven histogram distribution, whereas the cipher image shows a highly uniform histogram. This uniform pixel distribution verifies the favorable security performance of the proposed encryption scheme.

To further verify the pixel-distribution uniformity of encrypted images, this study employs the chi-square test for quantitative evaluation of encryption performance. As a classical statistical-analysis approach for distribution-consistency assessment, the $\chi^{2}$ test is particularly applicable to validating whether the pixel distribution of cipher images conforms to an ideal uniform distribution. The mathematical formula of the $\chi^{2}$ test is presented in Equation (42).

(42) $$\chi^{2}=\sum_{i=0}^{255}\frac{{\left(O_{i}-E_{i}\right)}^{2}}{E_{i}}$$

In the $\chi^{2}$ test model, $O_{i}$ denotes the observed pixel number corresponding to the *i*-th grayscale level, and *N* represents the total number of image pixels. Accordingly, $E_{i}=\frac{N}{256}$ is defined as the theoretical pixel count of each grayscale level under an ideal uniform distribution. In this statistical-evaluation experiment, a standard significance level of α=0.05 is set for hypothesis testing, and the degree of freedom corresponding to the pixel grayscale-level distribution test is determined to be 255. According to the standard chi-square distribution table and numerical calculation, the corresponding critical chi-square threshold under the above test conditions is 293.2478. The test judgment criterion is as follows: if the calculated chi-square statistical value of the encrypted image is less than the critical threshold, the null hypothesis is accepted, indicating that the pixel grayscale-value distribution of the encrypted image conforms to a uniform distribution in a statistical sense.

To fully validate the universal effectiveness and robustness of the proposed image encryption algorithm, comprehensive chi-square ($\chi^{2}$) statistical tests are conducted on a large number of test samples in this work. The test dataset covers multiple standard plaintext color images with different resolutions and distinct texture features; all corresponding ciphertext images generated by the proposed encryption scheme are included for comparative analysis. Quantitative statistical test results for all sample images are systematically summarized in Table 5. It can be clearly observed from the statistical data that the chi-square values of all tested encrypted images are significantly lower than the preset critical threshold of 293.2478, with no sample exceeding the threshold. These consistent experimental results fully demonstrate that the proposed encryption algorithm can thoroughly break the strong correlation and regular pixel-distribution rules existing in original plaintext images. The excellent pixel uniformity further proves that the proposed scheme possesses reliable anti-statistical-attack performance.

#### 5.4.2. Correlation Analysis of Adjacent Pixels

As a classic statistical tool, correlation analysis quantifies the correlation and similarity between datasets. In image-encryption research, it is widely adopted to assess the randomness and security of ciphertext images. Plaintext images typically exhibit strong correlation among adjacent pixels; this property may be exploited by adversaries for statistical cryptanalysis. Therefore, eliminating such neighboring-pixel correlation serves as an important security metric for image-encryption algorithms. The correlation coefficient is computed according to Equation (43):

(43) $$\left\{\begin{array}{cc} E(x) & =\frac{1}{N}\sum_{i=1}^{N}x_{i}\\ D(x) & =\frac{1}{N}\sum_{i=1}^{N}{\left(x_{i}-E\left(x\right)\right)}^{2}\\ \mathrm{cov}(x,y) & =\frac{1}{N}\sum_{i=1}^{N}\left(x_{i}-E\left(x\right)\right)\left(y_{i}-E\left(y\right)\right)\\ R_{xy} & =\frac{\mathrm{cov}(x,y)}{\sqrt{D(x)}\sqrt{D(y)}} \end{array}\right.$$

where *x* and *y* denote pixel-intensity sequences of adjacent pixels. cov(x,y) is the covariance, while D(x) and D(y) are the variances of *x* and *y*, respectively.

**Figure 10:**
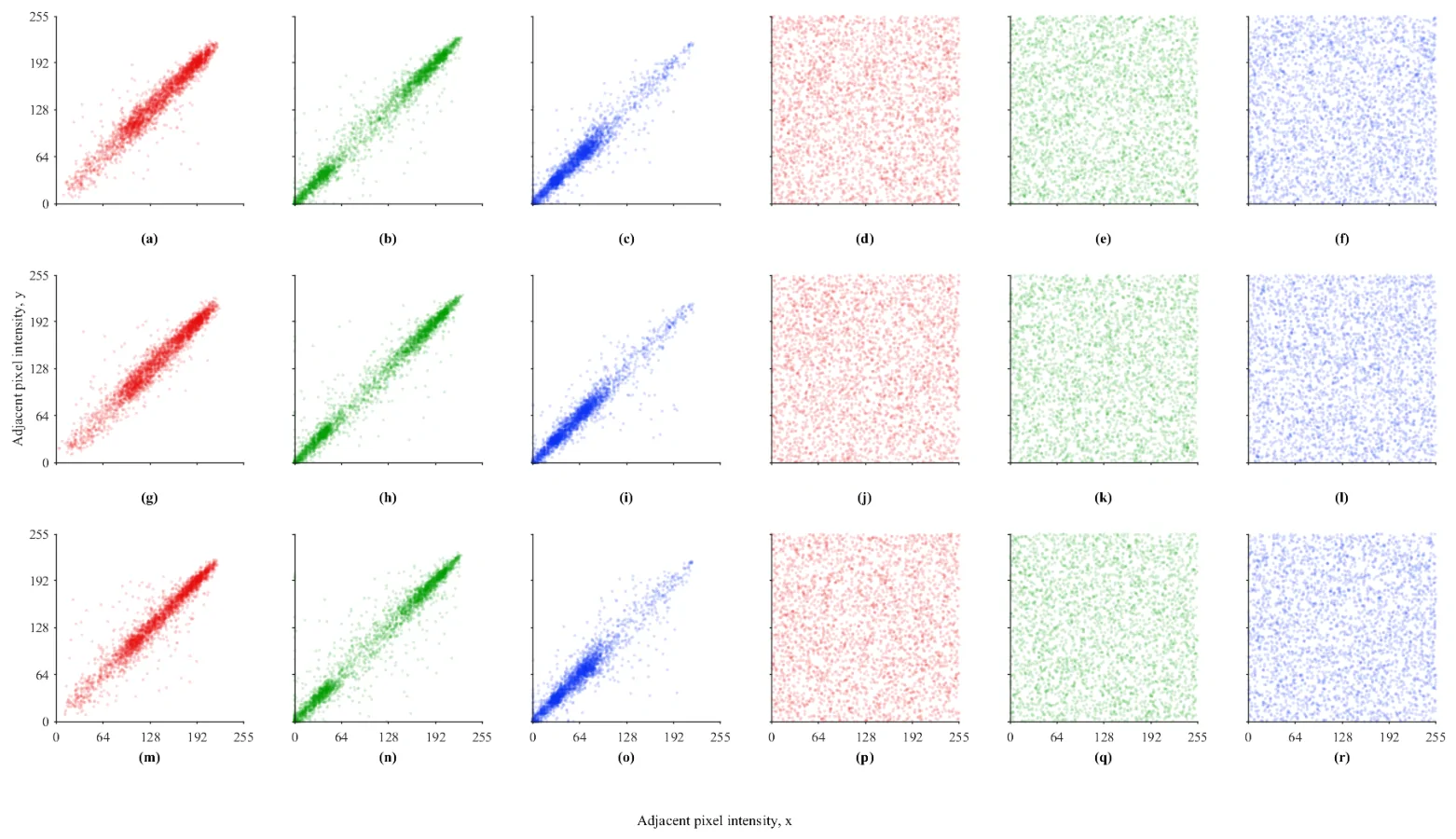
Pixel-pair distributions of the original and encrypted images in the horizontal, vertical, and diagonal directions: (**a**–**c**) original image, horizontal direction; (**d**–**f**) encrypted image, horizontal direction; (**g**–**i**) original image, vertical direction; (**j**–**l**) encrypted image, vertical direction; (**m**–**o**) original image, diagonal direction; (**p**–**r**) encrypted image, diagonal direction.

Figure 10 plots adjacent-pixel correlation distributions along horizontal, vertical, and diagonal directions for the plaintext and ciphertext of the *Peppers* image. Scatter plots for the R, G, and B channels of the plaintext exhibit obvious linear trends, which reflect strong spatial correlation between neighboring pixels. By contrast, ciphertext pixels are uniformly distributed without observable patterns, demonstrating that the proposed encryption effectively breaks the inherent spatial correlation among adjacent pixels.

As summarized in Table 6, the proposed algorithm produces near-zero correlation coefficients across all RGB channels for horizontal, vertical, and diagonal directions. More precisely, coefficients lie in the range of −0.0031 to 0.0018, and their absolute values are bounded by 0.0031. The mean absolute correlation coefficient over the nine channel-direction combinations equals 0.00132, outperforming the competing schemes from [37,38,39,40], whose respective mean values are 0.01628, 0.00484, 0.00278, and 0.00310. Even though some individual coefficients from competing methods are also close to zero, our scheme achieves the lowest overall correlation. These observations confirm that the proposed encryption suppresses spatial dependence between adjacent pixels and mitigates plaintext statistical-information leakage.

#### 5.4.3. Information Entropy

As an essential statistical metric, information entropy quantifies the randomness and complexity of images and has become a standard evaluation criterion for image encryption security. In an ideal encrypted image, the entropy of every color channel is nearly equal to the theoretical upper limit of 8, demonstrating outstanding unpredictability and strong resistance to various attacks. The computational formula for information entropy is presented in Equation (44):

(44) $$H\left(x\right)=\sum_{i=0}^{2^{n}-1}p\left(m_{i}\right)\log_{2}\frac{1}{p(m_{i})}$$

where $p(m_{i})$ represents the occurrence probability of symbol $m_{i}$. *n* is the number of bits required to represent message *m*, and $2^{n}$ corresponds to the total number of distinguishable symbols.

Information entropy is used to quantify the uncertainty of the ciphertext pixel-value distribution. For an 8-bit image channel, the theoretical maximum entropy is 8 bits. Table 7 reports the information entropy values of the R, G, and B channels for six encrypted test images with resolutions of 256×256 and 512×512.

All 18 channel-wise entropy values lie between 7.9975 and 7.9995, and the overall mean entropy is 7.9984. The largest deviation from the theoretical maximum is only 0.0025. In particular, consistently high entropy values are obtained for images with different textures, brightness distributions, and resolutions, indicating that the proposed scheme produces ciphertexts with stable randomness properties and a pixel-value distribution close to uniform.

These near-ideal entropy values suggest that the ciphertext reveals little statistical information about the plaintext intensity distribution and is therefore less susceptible to histogram-based statistical analysis. Together with the adjacent-pixel correlation results, the entropy analysis supports the statistical security of the proposed color-image encryption method.

To further evaluate the randomness and unpredictability of the proposed algorithm, we selected five representative state-of-the-art schemes from recent literature for comparative analysis. As shown in Table 8, the entropy values of the three channels are 7.9994, 7.9995, and 7.9995 for the proposed scheme, which are extremely close to the theoretical upper bound of 8.0. In terms of information entropy, the proposed method achieves superior randomness and cross-channel consistency over peer algorithms as the entropy values of red, green, and blue channels are close to the theoretical maximum of 8 and exhibit minor discrepancies across three channels, which reflects favorable statistical properties for image encryption.

### 5.5. Differential Attack

Differential cryptanalysis is a prevalent and effective attack method against image encryption schemes. It operates by imposing a minimal single-pixel perturbation on the original plaintext image and analyzing the resulting deviation of the ciphertext. Statistical analysis of these subtle changes enables adversaries to infer the internal operating mechanism and key information of the encryption system. Accordingly, an encryption algorithm that can generate significant ciphertext differences in response to trivial plaintext variations possesses robust resistance to differential attacks. To objectively quantify the plaintext sensitivity of encryption algorithms, NPCR and UACI are employed as standard evaluation indices. NPCR characterizes the proportion of varied pixels at corresponding positions of ciphertext images, whereas UACI quantifies the average magnitude of pixel differences. The theoretical ideal values of NPCR and UACI are 99.6094% and 33.4635%, respectively, and their mathematical formulas are given in Equation (45).

(45) $$\left\{\begin{array}{cc} D(i,j) & =\left\{\begin{array}{cc} 1, & C\left(i,j\right)\neq C^{\prime }\left(i,j\right),\\ 0, & \mathrm{otherwise}, \end{array}\right.\\ NPCR & =\frac{\sum_{i,j}D\left(i,j\right)}{m\times n}\times 100\%,\\ UACI & =\frac{1}{m\times n}\sum_{i,j}\frac{\left|C\left(i,j\right)-C^{\prime }\left(i,j\right)\right|}{255}\times 100\%. \end{array}\right.$$

In the calculation process, C(i,j) and $C^{\prime }\left(i,j\right)$ respectively represent the pixel value of the original encrypted image at position (i,j) and the pixel value of the encrypted image after a single-pixel perturbation at the same position, and m×n denotes the total number of pixels in the test image. Table 9 summarizes the theoretical reference ranges of NPCR and UACI for images with different sizes at a significance level of α=0.05, and these authoritative baseline data originate from the classic work by Wu and Noonan [44].

To evaluate the plaintext sensitivity and differential-attack resistance of the proposed image encryption algorithm, NPCR and UACI are utilized for quantitative security evaluation. Table 10 and Table 11 summarize the single-channel and average NPCR and UACI results on five standard test images, including 256×256-sized Airplane, House, Boats and Barbara, as well as 512×512-sized Baboon and Peppers. The diversified resolutions and texture features of these images facilitate a comprehensive evaluation of the algorithm’s generalization capability. For 8-bit RGB images, the theoretical optimal NPCR and UACI values are 99.6094% and 33.4635%, respectively. Experimental results reveal that our scheme achieves average NPCR ranging from 99.5715% to 99.6386% and UACI from 33.3319% to 33.5129% over all test images. All red, green, and blue channels satisfy the security thresholds. Such ideal and consistent metric values demonstrate that minor pixel perturbations in plaintext images can induce substantial changes in ciphertexts, which suggests favorable plaintext sensitivity and desirable statistical performance against differential attacks for the proposed algorithm.

For further validation of differential-attack resistance, the NPCR and UACI results of the proposed scheme are compared with those of three representative image-encryption methods on the standard Peppers image, as reported in Table 12 and Table 13. The proposed scheme achieves an average NPCR of 99.6111%, which differs from the theoretical value of 99.6094% by only 0.0017 percentage points, as does the scheme in [45]. Moreover, the NPCR values of the three color channels remain within the narrow interval from 99.6073% to 99.6187%, indicating that a one-pixel plaintext modification propagates to nearly all ciphertext pixels in each channel. For UACI, the proposed method obtains an average value of 33.4783%, which is close to the theoretical expectation of 33.4635%. Although some compared methods exhibit a slightly smaller deviation from the theoretical UACI value, the proposed scheme remains within the expected random-cipher range and outperforms the method in [41] in terms of proximity to the ideal UACI value. Taken together, the results demonstrate that the proposed scheme provides strong and competitive differential-attack resistance, with particularly near-optimal NPCR performance across the RGB channels.

### 5.6. Noise Attack Resistance

Note that the authentication check is manually bypassed in these noise-corruption experiments, as described in Section 5.1.

In the field of information encryption, noise interference is a common form of external disturbance, among which salt-and-pepper noise is one of the most typical threats to image encryption systems. This type of noise randomly generates discrete black-and-white pixel outliers within the encrypted image, severely degrading visual clarity and destroying image texture features. In practical transmission and application scenarios, qualified image encryption algorithms are required to possess robust anti-noise capability so that the decrypted image can effectively tolerate salt-and-pepper noise interference and recover plaintext information with minimal distortion.

**Figure 11:**
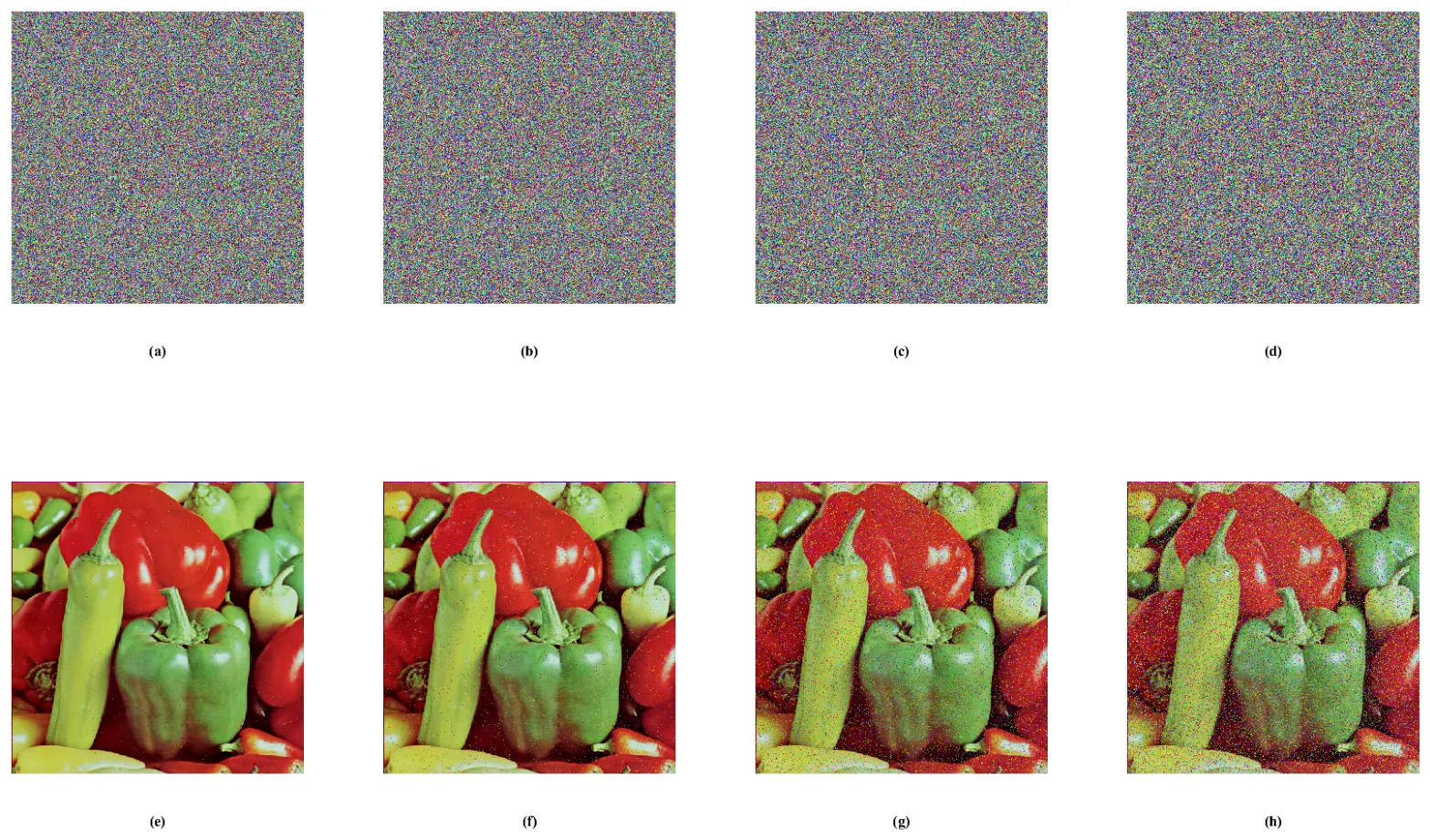
Salt-and-pepper noise attack simulation results. (**a**–**d**) Cipher images contaminated by salt-and-pepper noise with noise densities of 0.001, 0.01, 0.05 and 0.1, respectively. (**e**–**h**) Corresponding decrypted images.

**Figure 12:**
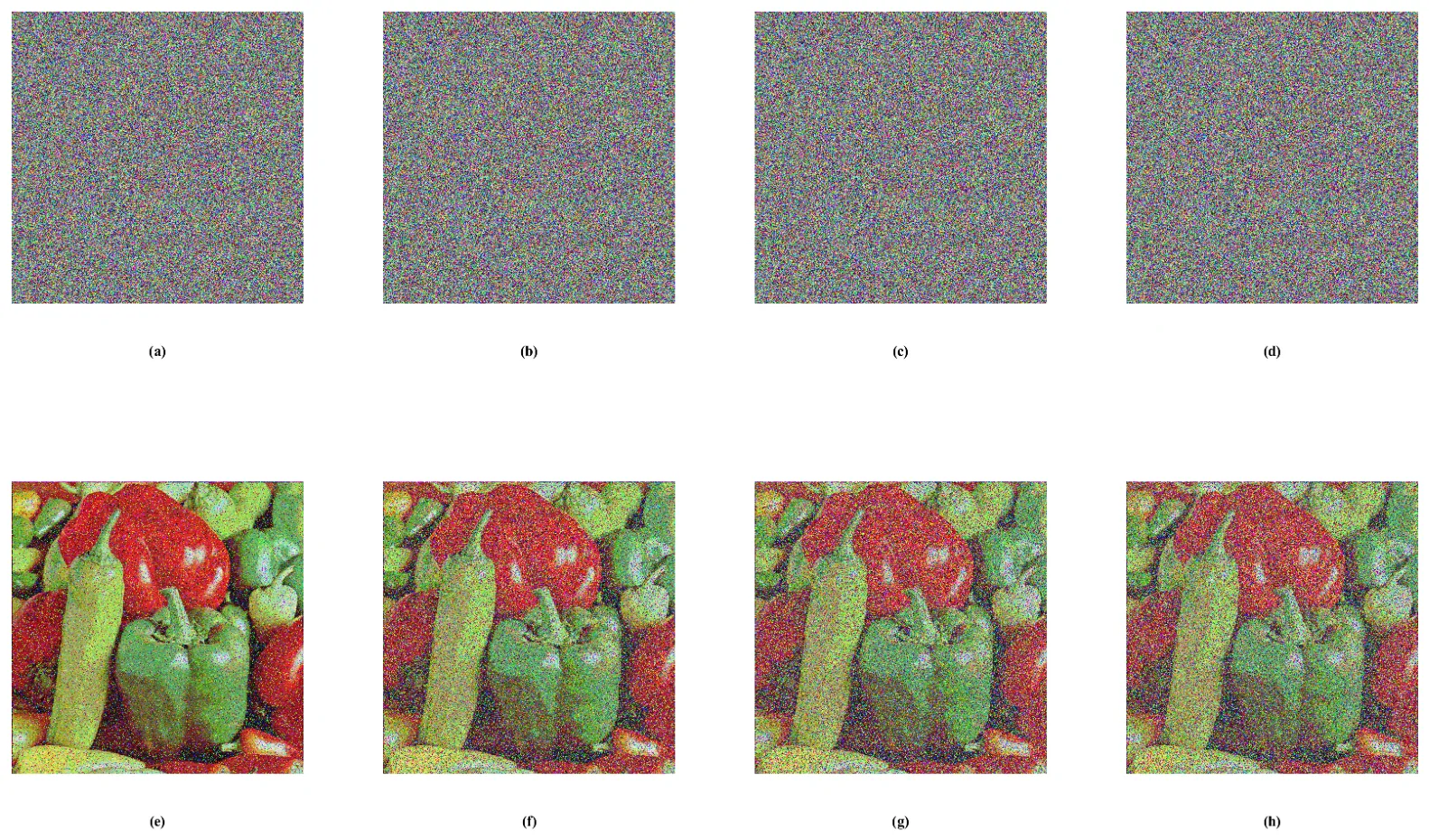
Gaussian noise attack test results. (**a**–**d**) Cipher images contaminated by Gaussian noise with variances of 0.001, 0.005, 0.008 and 0.01, respectively. (**e**–**h**) The corresponding decrypted images.

To fully validate the noise-resistance capability of the proposed encryption scheme, we conduct systematic noise-interference experiments on the standard Peppers test image. Two representative noise types in digital image processing, salt-and-pepper noise and Gaussian noise, are selected to simulate complex real-world interference environments, and multiple noise intensities are set for graded comparative tests. In the salt-and-pepper noise experiment, four progressive noise densities of 0.001, 0.01, 0.05, and 0.1 are superimposed on encrypted images to simulate different degrees of impulse-noise pollution. The corresponding visual results of decrypted images under varying noise intensities are demonstrated in Figure 11. Meanwhile, zero-mean Gaussian noise with four gradient variance levels of 0.001, 0.005, 0.008, and 0.01 is introduced into encrypted images to simulate continuous random-noise interference. The decrypted image outcomes under Gaussian-noise interference at different intensities are presented in Figure 12. These graded experimental settings cover slight, moderate, and severe noise-interference conditions, which can comprehensively reflect the stable anti-interference performance of the proposed algorithm in complex practical scenarios.

Visual observation indicates that noise artifacts in decrypted images gradually become prominent as noise intensity increases. Nevertheless, the proposed algorithm maintains favorable image recognizability under low- and medium-noise levels. Notably, the decrypted image retains clear visual features even when the salt-and-pepper noise density rises to 0.1. For Gaussian-noise interference, the image remains distinguishable at a variance of 0.005. Although further increasing Gaussian-noise intensity blurs partial image details, the overall structural contours can still be clearly identified. These intuitive phenomena preliminarily demonstrate the excellent robustness of the proposed scheme against severe noise interference.

To quantitatively assess the noise robustness of the proposed encryption scheme, peak signal-to-noise ratio (PSNR) and mean square error (MSE) are adopted as evaluation metrics. Specifically, a higher PSNR value corresponds to better fidelity of the decrypted image, while a lower MSE value indicates smaller pixel deviation between the original and decrypted images, reflecting superior image-restoration quality. The calculation formulas for PSNR and MSE are defined in Equation (46).

(46) $$\left\{\begin{array}{cc} MSE & =\frac{1}{m\times n}\sum_{i=1}^{m}\sum_{j=1}^{n}{\left(e_{ij}-f_{ij}\right)}^{2}\\ PSNR & =10\times \log_{10}\left(\frac{255^{2}}{MSE}\right) \end{array}\right.$$

where $e_{ij}$ denotes the pixel value of the original plaintext image, $f_{ij}$ represents the pixel value of the corresponding decrypted image, and *m* and *n* refer to the height and width of the test image, respectively.

The PSNR and MSE values of the decrypted image under different noise intensities are summarized in Table 14 and Table 15. Quantitative analysis shows that PSNR values decline gradually while MSE values increase steadily as noise intensity grows, which objectively reflects the negative impact of noise interference on image-restoration quality. More importantly, even under high-intensity noise perturbation, the decrypted images maintain acceptable visual quality and retain complete basic structural information. The combination of visual observation and numerical results fully verifies that the proposed image encryption algorithm possesses outstanding anti-noise performance and reliable fault-tolerant capability, demonstrating good practical applicability for complex noisy transmission environments.

### 5.7. Cropping Attack

As stated in Section 5.1, authentication verification is deliberately disabled for visualizing decryption outputs under cropped ciphertext.

During image transmission and storage, partial data loss often occurs due to channel interference, equipment failure, or malicious attacks, which severely degrades image decryption performance. To evaluate the robustness of the proposed image encryption algorithm against incomplete data, we perform cropping-attack experiments to simulate partial occlusion and data loss of encrypted images during transmission.

Taking the Peppers image as the test object, encrypted images are cropped with different area ratios. To improve experimental validity and better simulate real-world attack scenarios, the same regional cropping operation is applied to all R, G, and B channels to completely erase local ciphertext information within the selected region. Random cropping attacks are performed on ciphertext images, where the lost area fractions are set to 1%, 5%, 10%, and 20% of the total image area. This experimental setup comprehensively verifies the decryption and reconstruction capability of the algorithm under data-loss conditions ranging from slight to severe. All cropping positions are randomly selected, and the damaged ciphertext images are then decrypted. The four cropping fractions correspond to four attack-intensity levels: slight interference, moderate tampering, severe damage, and extreme data loss.

**Figure 13:**
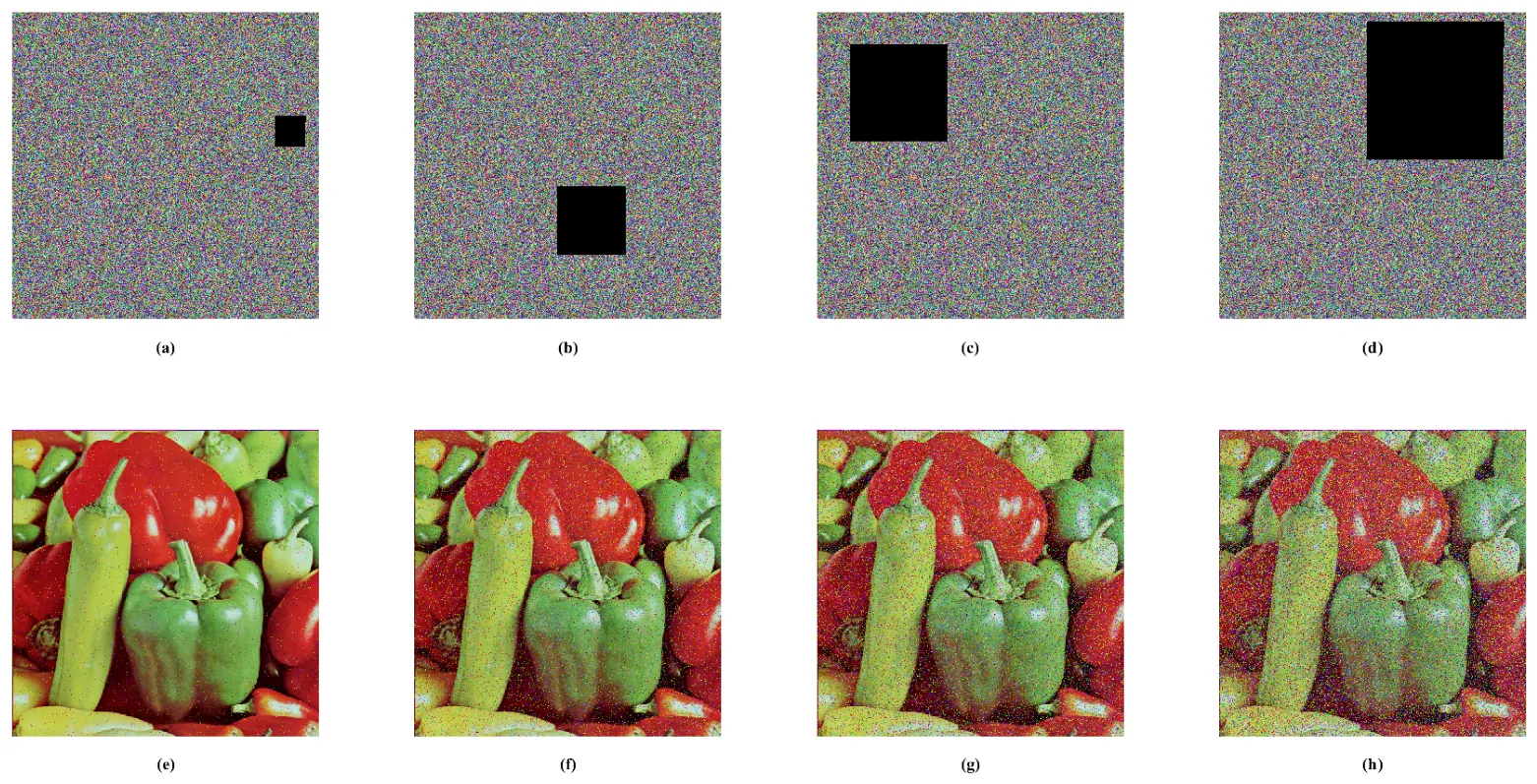
Results of the random-cropping attack. Ciphertext images subjected to random rectangular cropping with area-loss ratios of (**a**) 1%, (**b**) 5%, (**c**) 10%, and (**d**) 20%, respectively; (**e**–**h**) the corresponding decrypted images.

As illustrated in Figure 13, decrypted images retain satisfactory structural integrity and clarity under low cropping ratios, and the main image content can be accurately reconstructed. As the cropping area increases, the overall contour of the decrypted image remains recognizable, whereas fine details become increasingly blurred and partial local information suffers irreversible loss.

To further quantitatively analyze the algorithm’s anti-cropping performance, the peak signal-to-noise ratio (PSNR) and structural similarity index measure (SSIM) of decrypted images under different cropping ratios are calculated. The formula for SSIM calculation is defined in Equation (47):

(47) $$SSIM\left(x,y\right)=\frac{\left(2\mu_{x}\mu_{y}+C_{1}\right)\left(2\sigma_{xy}+C_{2}\right)}{\left(\mu_{x}^{2}+\mu_{y}^{2}+C_{1}\right)\left(\sigma_{x}^{2}+\sigma_{y}^{2}+C_{2}\right)}$$

where *x* and *y* represent pixel blocks of the decrypted image and the original image within a sliding window (default size: 11×11), respectively. $\mu_{x}$ and $\mu_{y}$ denote the mean pixel values of the two windows, $\sigma_{x}^{2}$ and $\sigma_{y}^{2}$ are the corresponding pixel variances, and $\sigma_{xy}$ refers to the covariance between the two windows. The parameters $C_{1}={\left(K_{1}L\right)}^{2}$ and $C_{2}={\left(K_{2}L\right)}^{2}$ are stability constants introduced to avoid division-by-zero errors, where $K_{1}=0.01$, $K_{2}=0.03$, and L=255 corresponds to the dynamic range of 8-bit grayscale images. The final SSIM value is computed as the arithmetic mean over all sliding windows, namely the mean SSIM (MSSIM), which ranges from [−1,1]. A value closer to 1 indicates higher structural similarity between the decrypted and original images.

Table 16 lists the variation trends of SSIM and PSNR values with increasing cropping ratio. As the cropping ratio rises, the structural integrity and pixel accuracy of the decrypted image gradually degrade, leading to an overall decrease in both SSIM and PSNR values. Under a low cropping fraction (1%), the decrypted image maintains relatively high SSIM and PSNR values, demonstrating the favorable performance of the proposed algorithm. The experimental results verify that the proposed encryption algorithm possesses excellent robustness against cropping attacks of varying severities. Under slight and moderate data-loss conditions, the algorithm can effectively reconstruct structural and detailed features of the original image, meeting the practical demands for secure and robust image transmission.

### 5.8. Hybrid Attack

Similar to noise and cropping tests, the HMAC authentication check is bypassed to observe decryption behavior under composite hybrid tampering.

**Figure 14:**
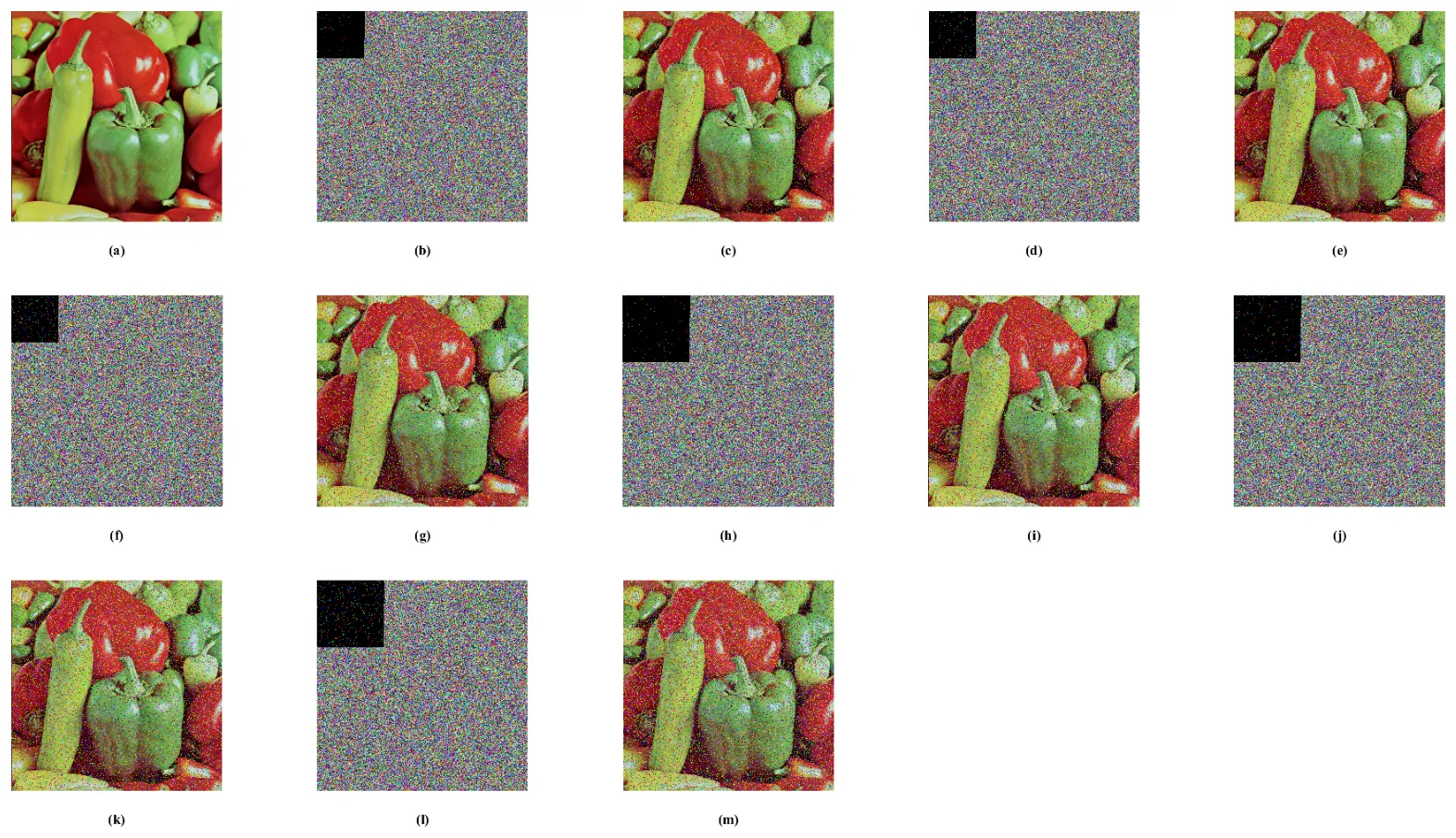
Hybrid-attack results under fixed rectangular cropping (i.e., removing a rectangular block) of the upper-left ciphertext region. (**a**) Original image. Panels (**b**,**d**,**f**) show ciphertext images subjected to 5.00% cropping and salt-and-pepper noise densities of 0.01, 0.02, and 0.05, respectively; panels (**c**,**e**,**g**) show the corresponding decrypted images. Panels (**h**,**j**,**l**) show ciphertext images subjected to 10.00% cropping and the same noise densities, respectively; panels (**i**,**k**,**m**) show the corresponding decrypted images.

To further validate the robustness of the proposed image encryption algorithm against complex adversarial scenarios, a hybrid cropping-noise attack experiment is designed to simulate practical composite attacks in real-world transmission environments. Different levels of spatial cropping and salt-and-pepper noise are superimposed to construct diverse hybrid attack conditions. In this experiment, the standard Peppers test image is adopted as the plaintext image. Two cropping ratios (5% and 10%) and three salt-and-pepper noise intensities (1%, 2%, and 5%) are set, forming a total of six hybrid attack scenarios. The corresponding encrypted images are decrypted, and the decryption quality is quantitatively evaluated using the peak signal-to-noise ratio (PSNR). Detailed experimental results are presented in Figure 14 and Table 17.

The experimental results demonstrate that decrypted images retain favorable visual quality and stable objective metrics even under severe hybrid attacks. Specifically, for a fixed cropping ratio of 5%, the PSNR value gradually decreases from 17.73 dB to 15.50 dB as the noise intensity increases from 1% to 5%. Although image details are disturbed by noise interference, the main structural features of the decrypted image remain clearly recognizable. When the cropping ratio increases to 10%, the PSNR value further declines to 13.98 dB under 5% noise interference. Nevertheless, the overall outline and color information of the original image can still be effectively recovered. These results show that the proposed algorithm can resist spatial information loss caused by cropping and pixel perturbation induced by noise. It is verified that the proposed encryption algorithm exhibits excellent robustness against composite hybrid attacks.

### 5.9. Chosen Plaintext Attack

In image encryption research, resistance against classical cryptanalysis attacks is a crucial criterion for evaluating the practical security of encryption algorithms. Typical attack models include the ciphertext-only attack (COA), known-plaintext attack (KPA), chosen-ciphertext attack (CCA), and chosen-plaintext attack (CPA). Among these attack types, CPA is widely regarded as the most powerful and challenging adversarial strategy. An encryption scheme that can effectively resist CPA is generally considered capable of withstanding the other three categories of classical attacks [43]. In real-world communication scenarios, adversaries may gain access to encryption equipment and arbitrarily generate plaintext-ciphertext pairs, which makes CPA a realistic security threat. In a CPA scenario, attackers can freely construct arbitrary plaintext images and obtain their corresponding ciphertexts, which allows them to explore potential mapping relationships between plaintext and ciphertext domains. The core threat of CPA is that attackers may deduce encryption keys or decrypt unknown ciphertexts by exploiting self-constructed plaintext-ciphertext pairs.

**Figure 15:**
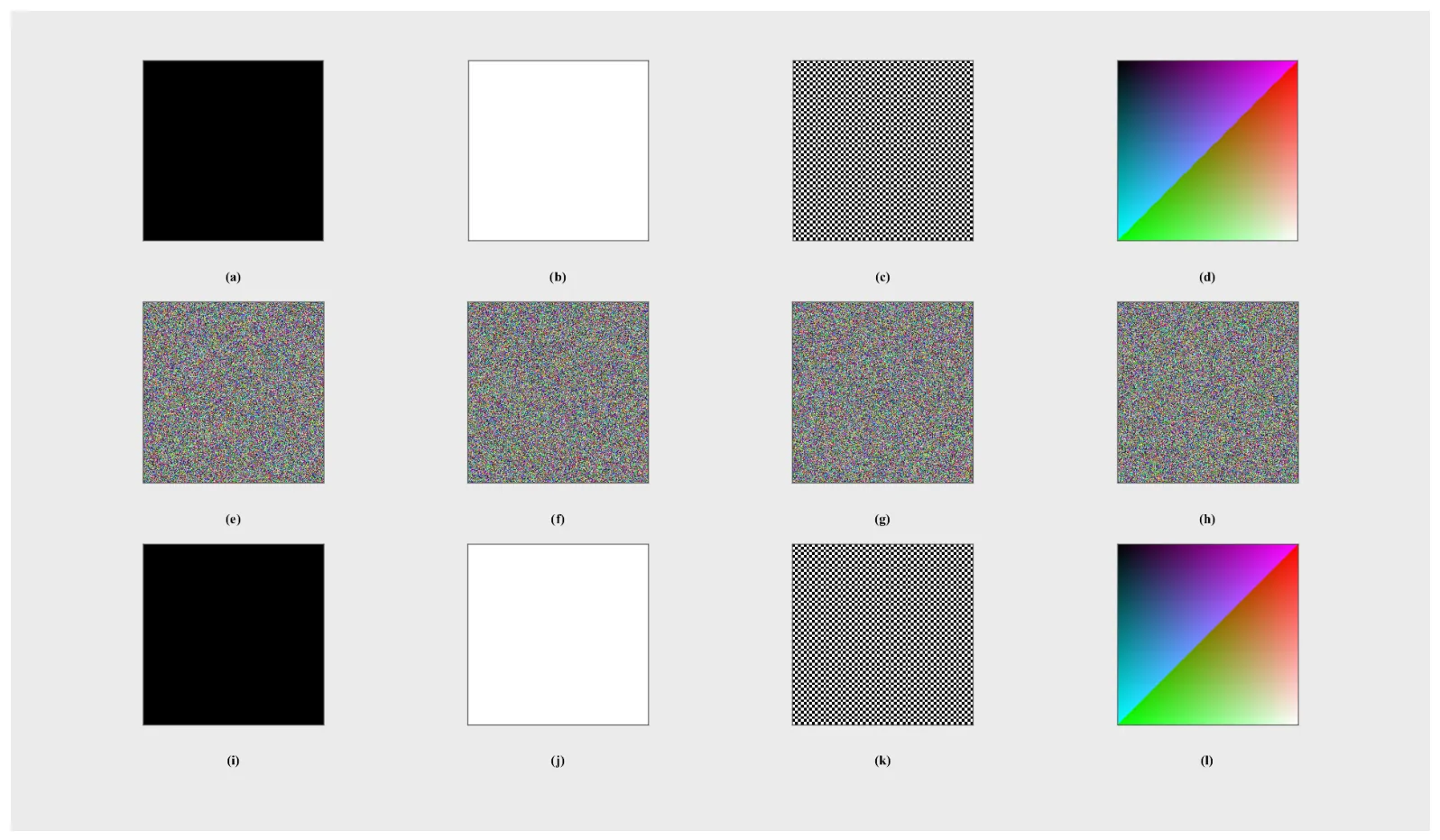
Visual results for the chosen-plaintext test: (**a**–**d**) all-black, all-white, checkerboard, and color-gradient plaintext images, respectively; (**e**–**h**) their corresponding ciphertext images; (**i**–**l**) the corresponding decrypted images.

To qualitatively evaluate the CPA resistance of the proposed image-encryption scheme, four representative plaintext images with distinct intensity distributions and spatial structures are selected: an all-black image, an all-white image, a checkerboard image, and a color-gradient image, as shown in Figure 15a–d. These inputs constitute challenging and highly distinguishable test patterns. Specifically, the all-black and all-white images exhibit uniform but opposite pixel distributions; the checkerboard image contains prominent periodic spatial features; and the color-gradient image presents smooth monotonic color transition across the image plane. Such deliberately constructed test images help verify whether plaintext features can be inferred from the resulting ciphertexts.

The corresponding encryption results are presented in Figure 15e–h. Although the four plaintext images differ greatly in visual appearance and statistical properties, their ciphertexts all show noise-like random distributions. No uniform dark or bright regions can be observed in ciphertexts generated from all-black and all-white inputs. Similarly, the periodic texture of the checkerboard image disappears after encryption, and the continuous color transition of the color-gradient image is completely destroyed. These observations demonstrate that the proposed scheme effectively masks spatial structures and color characteristics of the original plaintexts, preventing attackers from directly inferring plaintext content from ciphertexts. For completeness, the decrypted outputs are given in Figure 15i–l. Each decrypted image faithfully reproduces the content and structural features of its original plaintext, including uniform areas, checkerboard patterns, and smooth color gradients. This confirms the correctness and reversibility of the encryption-decryption procedure under valid secret parameters. Overall, the visual results provide qualitative evidence that the proposed algorithm hides salient plaintext features while enabling correct recovery for authorized users.

These observations preliminarily validate the favorable CPA resistance of the proposed encryption algorithm. Ciphertexts generated from different extreme plaintexts exhibit uniform random-noise characteristics and retain no valid plaintext features. Accordingly, attackers cannot extract effective rules or deduce key information from plaintext-ciphertext pairs. It further demonstrates that the proposed algorithm can defend against active adversarial attacks such as CPA and ensure reliable security for practical image transmission. To further consolidate this conclusion, information entropy is adopted as a quantitative metric to measure the randomness of ciphertexts produced under CPA conditions.

Table 18 lists the information entropy values of ciphertexts obtained under CPA tests. For the four extreme plaintext images (all-black, all-white, checkerboard, and color-gradient), the entropy values of red, green, and blue channels of their ciphertexts are very close to the theoretical maximum of 8, ranging from 7.9994 to 7.9995. Entropy remains high and stable with negligible fluctuations across different plaintext inputs and RGB channels. This indicates that the proposed encryption algorithm can effectively resist CPA. It thoroughly disrupts the intrinsic statistical features of plaintext images and produces ciphertexts with near-ideal randomness.

Our chosen-plaintext attack evaluation is an experimental simulation based on typical extreme test images. It provides empirical evidence of resistance but does not constitute a formal provable security proof. For practical deployment, the authenticated encryption mechanism (HMAC-SHA-256) is essential to reject manipulated ciphertext inputs.

### 5.10. Computational Complexity Analysis

The computational complexity of the proposed encryption scheme is analyzed for a single RGB image of size M×N. The analysis excludes image input/output and visualization operations. Let *s* denote the edge-extension width, and let $M_{p}$ and $N_{p}$ be the padded image dimensions. According to the implementation,

(48) $$M_{p}=8\left\lceil \frac{M+2s}{8}\right\rceil ,\;N_{p}=8\left\lceil \frac{N+2s}{8}\right\rceil .$$

Let

(49) $$P=M_{p}N_{p}$$

denote the number of pixels in one padded channel. Since the edge-extension width and the block size are bounded constants, P=Θ(MN).

During encryption, six principal chaotic sequences of length *P* are generated. Three sequences are employed for the initial permutation of the RGB channels, while the other three sequences are used to construct the key matrices. Since the transient iteration number $T_{0}=1000$ is fixed, the complexity of chaotic-sequence generation is O(P). Three additional short chaotic sequences are generated for the DNA initial vectors. Their computational cost is independent of *P* and therefore does not affect the asymptotic complexity.

Under the conventional comparison-based sorting model, the initial permutation requires sorting three chaotic sequences of length *P*, which yields a complexity of

(50) O(3PlogP).

Moreover, the two row-column permutation stages require four sorting operations. Their total complexity is

(51) $$O\left(2M_{p}\log M_{p}+2N_{p}\log N_{p}\right).$$

Edge processing, key-matrix construction, DNA encoding, DNA operations, diffusion, DNA decoding, plaintext hashing, and HMAC-based authentication process image or ciphertext bytes whose total amount is linear in *P*. Therefore, their total complexity is O(P). The overall encryption complexity is thus given by

(52) $$\begin{array}{ccc} T_{\mathrm{enc}} & = & O\left(3P\log P+2M_{p}\log M_{p}+2N_{p}\log N_{p}+P\right)\\ \; & = & O(P\log P). \end{array}$$

During decryption, the authentication tag is first verified. The algorithm then reconstructs the chaotic sequences and performs inverse row-column permutations, inverse DNA operations, inverse initial permutation, and image recovery. The same three global sorting operations and four row-column sorting operations are required. Therefore, the decryption complexity is

(53) $$\begin{array}{ccc} T_{\mathrm{dec}}\; & = & O\left(3P\log P+2M_{p}\log M_{p}+2N_{p}\log N_{p}+P\right)\\ \; & = & O(P\log P). \end{array}$$

The algorithm stores padded RGB channels, chaotic sequences, permutation indices, key matrices, and DNA intermediate results. Although the MATLAB implementation uses cell arrays to represent DNA symbols and consequently introduces a relatively large practical memory constant, the number of stored elements grows linearly with *P*. Hence, the space complexity is

(54) S=O(P).

Since P=Θ(MN), the time and space complexities of the proposed single-image encryption scheme can be expressed as

(55) $$T_{\mathrm{enc}}=T_{\mathrm{dec}}=O\left(MN\log(MN)\right),\;S=O\left(MN\right).$$

The computational complexity of the proposed image encryption scheme is shown in the Table 19.

## 6. Conclusions

To address the limitations of conventional image-encryption algorithms in structural randomness and resistance to cryptanalytic attacks, this paper proposes a color image-encryption scheme based on a novel chaotic map and DNA-domain chained operations. Specifically, a novel one-dimensional chaotic map, termed STLEM, is constructed by fusing sine, tent, logistic and exponential nonlinear components, which are equipped with boundary-value correction rules for mitigating finite-precision floating-point numerical degradation. We systematically analyze the influences brought by modulo operations, boundary clamping and NaN/inf reset rules on chaotic dynamics. The dynamical performance of STLEM is evaluated using Lyapunov exponent analysis, bifurcation diagrams, autocorrelation analysis, permutation entropy, approximate entropy, Lempel–Ziv complexity, the 0–1 test for chaos, and NIST SP 800-22 randomness tests, together with comparisons with representative one-dimensional chaotic maps. Compared with classic one-dimensional chaotic systems, STLEM delivers larger Lyapunov exponents, wider stable chaotic parameter ranges and better sequence randomness.

Based on STLEM, a complete authenticated color image-encryption framework is developed. The scheme employs a 256-bit master key, two independent chaos parameter groups, and HMAC-SHA-256-based key derivation. A plaintext hash, a cryptographically secure random salt, and a nonce are used to derive plaintext-aware chaotic initial states, while an HMAC authentication tag is employed to verify the integrity and authenticity of the ciphertext and public metadata before decryption. Different from static global DNA coding strategies, an adaptive position-dependent DNA encoding strategy is adopted, where each pixel block dynamically selects its coding rule from chaotic outputs to reduce potential plaintext frequency-leakage risks under partial permutation-layer compromise. The encryption process integrates dynamic redundant edge embedding, multi-stage chaotic pixel permutation, adaptive position-dependent DNA encoding, DNA-domain chained CBC-like diffusion, and two-stage row-column permutation.

Numerical simulations are carried out using fixed, reproducible secret parameters provided in Section 5.1. Experimental results demonstrate that the proposed scheme produces noise-like ciphertext images and enables lossless image recovery with correct secret inputs. The scheme exhibits a large key space, near-ideal information entropy, negligible adjacent-pixel correlation, and satisfactory NPCR and UACI values. Additional experiments further validate its robustness against noise interference, data cropping and hybrid composite attacks. In addition, the authentication mechanism prevents normal decryption when the ciphertext, metadata, master key, or chaos parameters are incorrect. The proposed method has a computational complexity of O(MNlog(MN)) and a space complexity of O(MN) for a single image of size M×N.

We also discuss the practical limitations of the presented approach. Similar to all digital chaotic cryptosystems, finite-precision floating-point orbit-shortening cannot be fully eliminated, although HMAC-based seed derivation and transient-iteration discarding are applied as mitigation measures. Moreover, our MATLAB-based prototype yields relatively high computational overhead when compared against highly-optimized standard block-cipher implementations such as AES.

When benchmarked against existing chaos-DNA color-image encryption works, our implementation has three distinct strengths. Firstly, the STLEM map supports wider stable chaotic parameter domains and superior randomness metrics relative to many traditional one-dimensional chaotic maps in existing literature. Secondly, position-dependent adaptive DNA encoding is utilized instead of fixed global DNA mapping rules, which alleviates plaintext frequency-leakage hazards if permutation layers are partially broken. Thirdly, native authenticated encryption based on HMAC-SHA-256 is tightly integrated for seed generation and ciphertext verification—a feature missing in most comparable chaos-DNA implementations—so tampered ciphertext and invalid keys can be rejected prior to decryption.

For comparison with the non-chaotic standard block cipher AES, AES-GCM is a mature hardware-accelerated primitive with well-studied provable-security assumptions, yet it treats image content as generic byte streams and lacks image-oriented optimization. Under AES-CBC mode, corrupted ciphertext blocks will produce completely garbled decrypted output. Benefiting from dynamic redundant-edge expansion, multi-stage spatial scrambling and DNA-domain chained diffusion, our cryptosystem achieves better visual recoverability under noise and cropping-induced data loss. Nevertheless, such improved robustness comes with extra computational cost. This algorithm targets specialized robust color-image communication scenarios and is not intended as a universal drop-in substitute for AES.

Future work will focus on computational-efficiency optimization, deeper formal cryptanalysis for the STLEM-based encryption pipeline, as well as hardware implementation for real-time secure color-image communication applications.

## Figures and Tables

**Table 1:** Reproducible protocol for the STLEM 0–1 test.

Item	Setting
Parameter vectors	68: four manually defined reference vectors and 64 scrambled Sobol quasi-random samples
Parameter sampling domain	a∈[0.0,9.0], b∈[−1.6,2.0], c∈[−0.5,0.5], and d∈[−2.0,0.0]
Initial conditions	Four predefined initial values evaluated for each parameter vector
Transient and retained length	5000 transient iterations discarded; $N_{s}=10,000$ orbit points retained for analysis
Frequency set	100 equally spaced interior samples within [π/5,4π/5]
Lag range	$n=1,\ldots ,n_{\mathrm{cut}}$, with $n_{\mathrm{cut}}=1000=\left\lfloor N_{s}/10\right\rfloor$
Representative frequency	θ=2.17734
Precision and boundary handling	IEEE-754 double precision; reset boundary policy activated; zero correction events recorded across all 272 trajectories

**Table 2:** NIST SP-800-22 statistical test results.

NIST Statistical Test Item	*p*-Value	Pass Rate	Results
Frequency (monobit)	0.657933	99/100	Pass
Block Frequency (m=128)	0.494392	100/100	Pass
Cumulative Sums (Forward)	0.366918	99/100	Pass
Cumulative Sums (Reverse)	0.678686	99/100	Pass
Runs	0.236810	100/100	Pass
Longest Run of Ones	0.657933	100/100	Pass
Rank	0.035174	100/100	Pass
FFT	0.798139	99/100	Pass
Non-Overlapping Templates (m=9)	0.699313	99/100	Pass
Overlapping Templates (m=9)	0.534146	99/100	Pass
Universal	0.759756	100/100	Pass
Approximate Entropy (m=10)	0.249284	99/100	Pass
Random-Excursions (X=4)	0.383827	58/58	Pass
Random-Excursions Variant (X=−9)	0.699313	58/58	Pass
Serial Test 1 (m=16)	0.319084	99/100	Pass
Serial Test 2 (m=16)	0.739918	99/100	Pass
Linear Complexity	0.637119	98/100	Pass

**Table 3:** Eight DNA coding rules satisfying the Watson–Crick complementarity constraint.

DNA Base	Rule 1	Rule 2	Rule 3	Rule 4	Rule 5	Rule 6	Rule 7	Rule 8
A	00	00	01	10	01	10	11	11
T	11	11	10	01	10	01	00	00
C	01	10	00	00	11	11	01	10
G	10	01	11	11	00	00	10	01

**Table 4:** DNA arithmetic operations. Entries are reported in the order $O_{1}/O_{2}/O_{3}/O_{4}/O_{5}$.

	A	G	C	T
A	A/A/A/G/T	G/T/G/C/C	C/C/C/T/G	T/G/T/A/A
G	G/G/G/C/A	C/A/A/T/T	T/T/T/A/C	A/C/C/G/G
C	C/C/C/T/G	T/G/T/A/A	A/A/A/G/T	G/T/G/C/C
T	T/T/T/A/C	A/C/C/G/G	G/G/G/C/A	C/A/A/T/T

**Table 5:** $\chi^{2}$ test results for plaintext and ciphertext images.

Image	Size	Channel	χ^2^ Test Results	
			Plaintext	Ciphertext
Airplane	256×256	R	153,618.91	285.37
		G	150,590.67	252.20
		B	256,176.53	265.05
House	256×256	R	258,576.88	222.84
		G	299,158.64	241.48
		B	394,038.95	238.93
Boats	256×256	R	70,299.32	256.74
		G	106,665.40	232.55
		B	132,823.05	246.76
Barbara	256×256	R	25,461.66	232.37
		G	40,543.54	277.53
		B	35,054.88	249.56
Baboon	512×512	R	87,774.62	264.95
		G	150,466.50	218.90
		B	103,306.20	235.36
Peppers	512×512	R	214,262.78	282.96
		G	375,296.41	227.32
		B	587,904.42	229.51
Passed/Total				18/18

**Table 6:** Correlation comparison of Peppers image encrypted by different algorithms.

Method	Channel	Correlation Coefficient		
		Horizontal	Vertical	Diagonal
Proposed	R	−0.0013	−0.0010	0.0027
	G	0.0007	−0.0020	0.0018
	B	−0.0011	0.0006	−0.0031
Ref. [37]	R	−0.0088	0.0088	0.0076
	G	0.0073	0.0326	0.0048
	B	−0.0186	0.0362	0.0217
Ref. [38]	R	−0.0091	−0.0059	−0.0011
	G	0.0039	0.0010	−0.0079
	B	0.0073	−0.0067	0.0047
Ref. [39]	R	0.0007	−0.0040	0.0052
	G	−0.0066	0.0039	−0.0037
	B	0.0006	0.0030	−0.0041
Ref. [40]	R	0.0003	−0.0039	0.0053
	G	0.0073	0.0029	0.0060
	B	0.0007	0.0008	0.0070

**Table 7:** Information entropy of encrypted images.

Image	Size	Encrypted Image		
		Red	Green	Blue
Airplane	256×256	7.9978	7.9979	7.9977
House	256×256	7.9977	7.9975	7.9977
Boats	256×256	7.9980	7.9982	7.9981
Barbara	256×256	7.9982	7.9978	7.9981
Baboon	512×512	7.9994	7.9995	7.9995
Peppers	512×512	7.9994	7.9995	7.9995

**Table 8:** Information entropy comparison of encrypted images using different algorithms.

Method	Encrypted Image		
	Red	Green	Blue
Proposed	7.9994	7.9995	7.9995
[37]	7.9982	7.9985	7.9984
[38]	7.999	7.999	7.999
[41]	7.9992	7.9993	7.9993
[42]	7.9982	7.9982	7.9981
[43]	7.9993	7.9993	7.9993

**Table 9:** Theoretical NPCR and UACI values for different image sizes.

Size	NPCR (%)	UACI (%)	
		UACI−	UACI+
256×256	99.5693	33.2824	33.6447
512×512	99.5893	33.3730	33.5541
1024×1024	99.5994	33.4183	33.5088

**Table 10:** Differential Attack: NPCR (%).

Img	Size	R	G	B	Average
Airplane	256×256	99.6040	99.6040	99.6180	99.6086
House	256×256	99.6386	99.6159	99.6082	99.6209
Boats	256×256	99.6310	99.5996	99.6148	99.6151
Barbara	256×256	99.5910	99.5715	99.6061	99.5895
Baboon	512×512	99.6172	99.6272	99.6196	99.6213
Peppers	512×512	99.6073	99.6187	99.6073	99.6111
Pass/All		6/6	6/6	6/6	6/6

**Table 11:** Differential Attack: UACI (%).

Img	Size	R	G	B	Average
Airplane	256×256	33.5293	33.3692	33.5058	33.4681
House	256×256	33.3319	33.3891	33.4555	33.3922
Boats	256×256	33.3929	33.4083	33.5076	33.4362
Barbara	256×256	33.4723	33.4953	33.4412	33.4696
Baboon	512×512	33.4461	33.4402	33.4789	33.4551
Peppers	512×512	33.4625	33.4596	33.5129	33.4783
Pass/All		6/6	6/6	6/6	6/6

**Table 12:** NPCR (%) comparison on Peppers image with different algorithms.

Method	Red	Green	Blue	Average
Proposed	99.6073	99.6187	99.6073	99.6111
[41]	99.62	99.62	99.63	99.6233
[43]	99.62	99.61	99.61	99.6133
[45]	99.6139	99.6185	99.6010	99.6111

**Table 13:** UACI (%) comparison on Peppers image with different algorithms.

Method	Red	Green	Blue	Average
Proposed	33.4625	33.4596	33.5129	33.4783
[41]	33.54	33.47	33.47	33.5167
[43]	33.48	33.44	33.46	33.46
[45]	33.4550	33.5032	33.4562	33.4715

**Table 14:** PSNR and MSE values of decrypted images under different salt-and-pepper noise densities.

Index	0.001	0.01	0.05	0.1
PSNR	34.64	24.93	18.08	15.12
MSE	22.3607	209.1348	1012.0857	2001.8452

**Table 15:** PSNR and MSE values of decrypted images under different Gaussian noise variances.

Index	0.001	0.005	0.008	0.01
PSNR	14.81	11.95	11.19	10.83
MSE	2148.5792	4153.7009	4945.6514	5370.2138

**Table 16:** PSNR and SSIM values of Peppers decrypted images under different cropping ratios.

Index	1%	5%	10%	20%
PSNR	25.04	18.47	15.60	12.95
SSIM	0.7290	0.3333	0.1936	0.1115

**Table 17:** PSNR values of Peppers decrypted images under hybrid cropping-noise attack conditions. Columns denote: C5N1 (5% cropping + 1% noise), C5N2 (5% cropping + 2% noise), C5N5 (5% cropping + 5% noise), C10N1 (10% cropping + 1% noise), C10N2 (10% cropping + 2% noise), C10N5 (10% cropping + 5% noise).

Attack Condition	C5N1	C5N2	C5N5	C10N1	C10N2	C10N5
PSNR (dB)	17.73	17.03	15.50	15.28	14.91	13.98

**Table 18:** Information entropy of ciphertext images under chosen-plaintext attacks.

Image	Red	Green	Blue
All-black image	7.9994	7.9995	7.9994
All-white image	7.9994	7.9994	7.9994
Checkerboard image	7.9995	7.9994	7.9994
Color-gradient image	7.9994	7.9994	7.9995

**Table 19:** Computational complexity of the proposed image encryption scheme.

Operation	Encryption	Decryption
Chaotic-sequence generation	O(P)	O(P)
Global pixel permutation	O(3PlogP)	O(3PlogP)
Row-column permutations	$O(M_{p}\log M_{p}+N_{p}\log N_{p})$	$O(M_{p}\log M_{p}+N_{p}\log N_{p})$
DNA operations and diffusion	O(P)	O(P)
Hashing and authentication	O(P)	O(P)
Overall time complexity	O(PlogP)	O(PlogP)
Space complexity	O(P)	O(P)

## Data Availability

The original contributions presented in this study are included in the article/Supplementary Material. Further inquiries can be directed to the corresponding author.

## Supplementary Materials

The following supporting information can be downloaded at https://www.mdpi.com/article/10.3390/e28091015/s1. Algorithm S1: STLEM chaotic map iteration function; Algorithm S2: DNA pixel encoding function; Algorithm S3: DNA arithmetic operation $O_{k}$; Algorithm S4: Get inverse DNA operation index for decryption.
