# Supplementary material for: A Color Image Encryption Scheme Using an Enhanced One-Dimensional Chaotic Map and Adaptive DNA Encoding
Source: Entropy (Basel). 2026 Sep 11;28(9):1015. doi: 10.3390/e28091015 (PMC13605838; doi:10.3390/e28091015)
Supplement: Supplementary file 1 [file entropy-28-01015-s001.zip › entropy-4515563-supplementary.pdf]

Supplementary Algorithms for "A Color Image Encryption Scheme Using an Enhanced One-Dimensional Chaotic Map and Adaptive DNA Encoding"

September 2, 2026

Algorithm S1 STLEM chaotic map iteration function

Require: x_{prev}: previous state; a, b, c, d: control parameters
Ensure: x_{next}: next chaotic state

$x \leftarrow \mathrm{mod}(x_{prev}, 1)$
if $\mathrm{isnan}(x) \vee \mathrm{isinf}(x)$ **then**
    $x \leftarrow 0.5$
end if
if $x \leq 0.5$ **then**
    $y \leftarrow \mathrm{mod}\big(ax + (8-a)\sin(\pi x) + bx^3 + ce^{dx},\ 1\big)$
else
    $y \leftarrow \mathrm{mod}\big(a(1-x) + (8-a)\sin(\pi(1-x)) + b(1-2x)^3 + ce^{d(1-x)},\ 1\big)$
end if
if $y < 10^{-12}$ **then**
    $y \leftarrow 0.48$
else if $y > 1 - 10^{-12}$ **then**
    $y \leftarrow 0.52$
end if
return y

Algorithm S2 DNA pixel encoding function

Require: $pixel \in [0, 255]$, $rule \in \{1, \ldots, 8\}$
Ensure: 4-symbol DNA character sequence

$byte \leftarrow \text{uint8}(pixel)$
split $byte$ into four 2-bit segments b_1, b_2, b_3, b_4
look-up table from Table 2 according to $rule$
$dna\_str \leftarrow$ map $[b_1, b_2, b_3, b_4]$ to DNA bases
return dna_str

Algorithm S3 DNA arithmetic operation $\mathcal{O}_k$

Require: $dnaX$, $dnaY$: 4-base DNA strings; $op\_idx \in \{1, 2, 3, 4, 5\}$
Ensure: output 4-base DNA string

map each DNA character to integer $\phi(\cdot) \in \{0, 1, 2, 3\}$
for $i = 1$ **to** 4 **do**
    $n_1 \leftarrow \phi(dnaX(i))$, $n_2 \leftarrow \phi(dnaY(i))$
    if $op\_idx = 1$ **then**
        $n_r \leftarrow \text{mod}(n_1 + n_2, 4)$
    else if $op\_idx = 2$ **then**
        $n_r \leftarrow \text{mod}(n_1 - n_2, 4)$
    else if $op\_idx = 3$ **then**
        $n_r \leftarrow n_1 \oplus n_2$
    else if $op\_idx = 4$ **then**
        $n_r \leftarrow \text{mod}(n_1 + n_2 + 1, 4)$
    else if $op\_idx = 5$ **then**
        $n_r \leftarrow \text{mod}(n_1 - n_2 - 1, 4)$
    end if
    $res(i) \leftarrow \phi^{-1}(n_r)$
end for
return res

Algorithm S4 Get inverse DNA operation index for decryption

Require: forward operation index op
Ensure: inverse operation index op_{inv}

mapping: $1 \leftrightarrow 2,\ 3 \leftrightarrow 3,\ 4 \leftrightarrow 5$
return corresponding inverse index
